# The Effects of Thermal Radiation on an Unsteady MHD Axisymmetric Stagnation-Point Flow over a Shrinking Sheet in Presence of Temperature Dependent Thermal Conductivity with Navier Slip

**DOI:** 10.1371/journal.pone.0138355

**Published:** 2015-09-28

**Authors:** Sabyasachi Mondal, Nageeb A. H. Haroun, Precious Sibanda

**Affiliations:** University of KwaZulu-Natal, School of Mathematics, Statistics and Computer Science, Private Bag X01, Scottsville, Pietermaritzburg 3209, South Africa; Abdul Wali Khan University Mardan Pakistan, PAKISTAN

## Abstract

In this paper, the magnetohydrodynamic (MHD) axisymmetric stagnation-point flow of an unsteady and electrically conducting incompressible viscous fluid in with temperature dependent thermal conductivity, thermal radiation and Navier slip is investigated. The flow is due to a shrinking surface that is shrunk axisymmetrically in its own plane with a linear velocity. The magnetic field is imposed normally to the sheet. The model equations that describe this fluid flow are solved by using the spectral relaxation method. Here, heat transfer processes are discussed for two different types of wall heating; (a) a prescribed surface temperature and (b) a prescribed surface heat flux. We discuss and evaluate how the various parameters affect the fluid flow, heat transfer and the temperature field with the aid of different graphical presentations and tabulated results.

## 1 Introduction

The study of an unsteady fluid flow toward a stretching/shrinking sheet has great importance due to its various applications in science and engineering. Some often given examples in this regard include metal rolling, drawing and pultrusion. Heat transfer in such flows with both constant and variable wall temperature was investigated by Gupta and Gupta [1] and also investigated by Carragher and Crane [2]. Work on unsteady MHD flow with ramped wall temperature has been done by Khan et al.[3], Samiulhaq [4] and Khalid [5]. Wang [6] investigated the steady flow through a flat surface of a viscous fluid which is stretched in its own plane in two perpendicular directions. MHD free convection of unsteady flow in a porous medium with Newtonian heating and constant mass diffusion was studied by Hussanan [7]. Pavlov [8] studied exact similarity solution of the steady two-dimensional boundary layer flow equations in presence of magnetic field of an electrically conducting fluid due to the stretching of an elastic surface in the presence of a uniform transverse magnetic field. Mabood et al. [9] solved the differential equations of the model flow and heat transfer in an axisymmetric channel using the optimal homotopy asymptotic method. Homann [10] studied three dimensional axisymmetric stagnation-point flow using a similarity transform for reducing the Navier-Stokes equations to third order ordinary differential equations.

Chiam [11] investigated steady axisymmetric stagnation-point flow of a viscous fluid over an axisymetrically stretched surface. Mahapatra and Gupta [12] examined axisymmetric stagnation-point flow of an incompressible viscous fluid towards a stretching surface. Axisymmetric stagnation-point flow in presence of a uniform magnetic field towards a stretching surface with heat generation was investigated by Attia [13].

Considerable interest has been shown on the boundary layer flow over a shrinking sheet in recent years. Some of the applications of the shrinking sheet problem in industry relate to the shrinking film that is can be unwrapped easily with adequate heat and used in the packaging of bulk products. The shrinking fluid flow study, which is essentially a backward flow, can also be applied to the study of hydraulic properties of agricultural clay soils, capillary effects in the shrinking-swell behaviour and small pores. The related changes in mechanical and hydraulic studies of such soils have a significant impact on the behaviour and the transport properties of the fluid. The fluid loses the memory of the perturbation produced by the slot for this backward flow configuration. Due this reason, the fluid flow due to a shrinking sheet has some quite distinct physical characteristics compared to the forward stretching case.

Miklavcic and Wang [14] studied axisymmetric flow with uniform suction induced by a shrinking surface. Wang [15] examined heat transfer from a shrinking sheet due to a steady two-dimensional axisymmetric stagnation-point flow. Qasim et al. [16] examined heat transfer in the case of a micropolar fluid through a stretching sheet with Newtonian heating. Recently, Mahapatra and Nanday [17] studied heat transfer in an axisymmetric stagnation-point flow in the presence of a magnetic field. Qayyum et al. [18] presented an analysis of unsteady axisymmetric squeezing fluid flow with slip boundary conditions through a porous channel. Some recent studies of boundary layer flow in presence of a magnetic field include those of Mabood and his group [19–21]. For the case of a nonlinearly stretching sheet, we note the work of Khan et al. [22].

In this paper, we generalize the study of MHD fluid flow with an unsteady conditions through a shrinking sheet including a temperature dependent thermal conductivity, radiation and a Navier slip condition. The surface with prescribed surface temperature (PST) and surface with prescribed wall heat flux (PHF) are considered as two examples of non-isothermal boundary conditions.

**Ethical Statement:** This study involved only numerical simulations and the analysis of fluid flow.

## 2 Formulation

**Fig 1:**
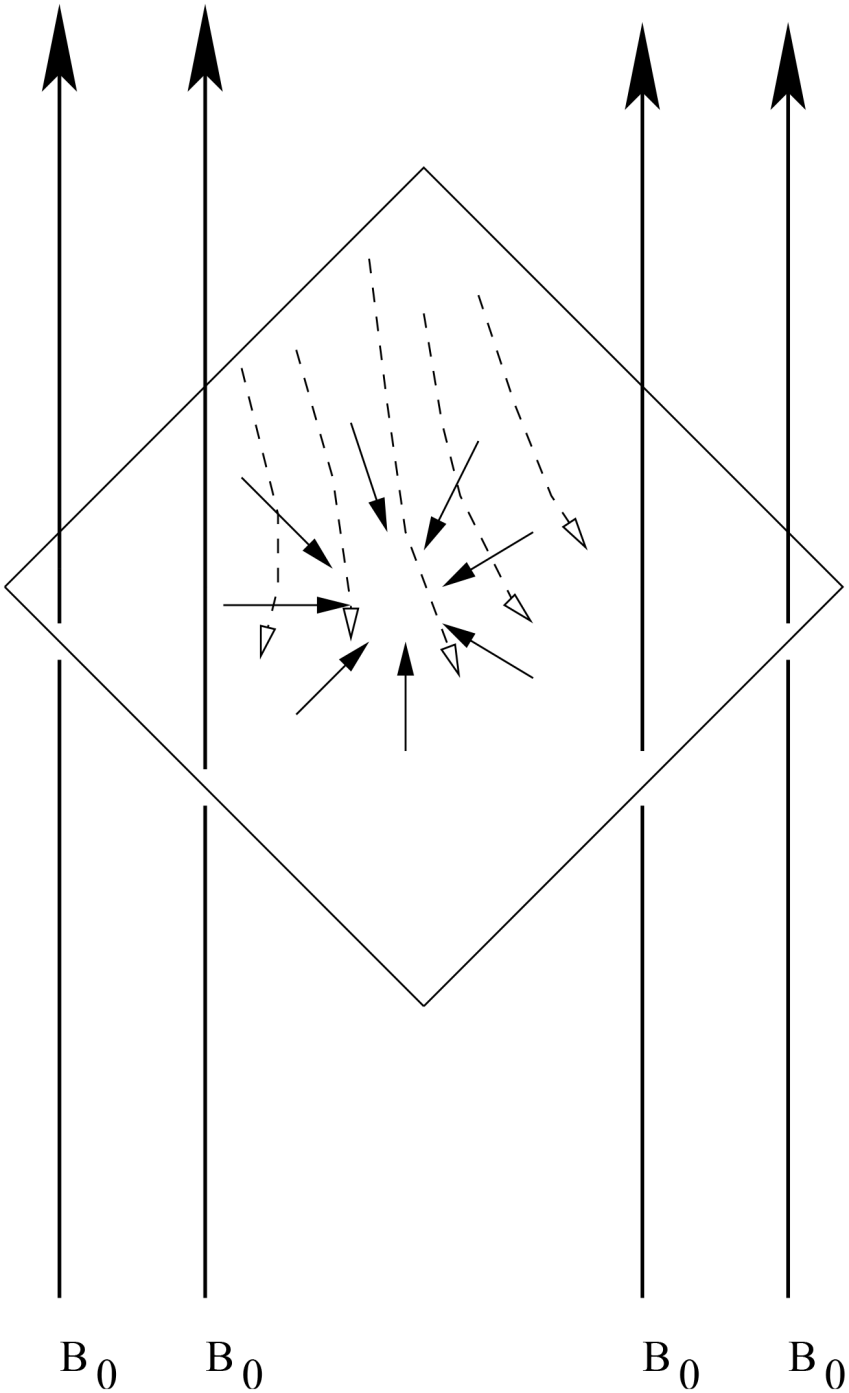
A sketch of the physical problem.

Here, we consider the unsteady axisymmetric stagnation-point flow of an electrically conducting incompressible fluid from the surface which is shrunk axisymmetrically. We have used Cartesian axes instead of cylindrical axes due to possible non-alignment, Wang [15]. The flow configuration is shown in Fig 1. In this frame of reference, let the velocity components are *u*, *v* and *w* in the *x*- direction, *y*- direction and *z*-direction, respectively.

At the surface the fluid velocity components are

$$u=\frac{(l+x)c}{1-\lambda t},\;v=\frac{cy}{1-\lambda t}\;\text{and}\;w=0,$$

where −*l* is the location of the origin and *c* (< 0) denotes the shrinking rate (and if *c* > 0 the it denotes stretching rate). Here the sheet shrinkage is along the negative *x*-axis. Note that the stretching axis and the point flow are not always aligned (that is, *l* ≠ 0).

The velocity components in the ambient region are given by

$$U=\frac{ax}{1-\lambda t},\;V=\frac{ay}{1-\lambda t}\;\text{and}\;W=-\frac{2az}{1-\lambda t},$$

where *a* (> 0) is a constant that is a measure of the strength of the stagnation-point flow and *λ* quantifies the unsteadiness of the problem. For a decelerating shrinking sheet *λ* < 0 whereas for an accelerating sheet *λ* > 0. The magnetic field *B*_0_ is imposed in the normal direction to the surface i.e., parallel to *z*-axis.

The continuity and momentum equations are (Bansal [23])

$$\begin{array}{c} \frac{\partial u}{\partial x}+\frac{\partial v}{\partial y}+\frac{\partial w}{\partial z}=0, \end{array}$$ (1)

$$\begin{array}{c} \frac{\partial u}{\partial t}+u\frac{\partial u}{\partial x}+w\frac{\partial u}{\partial z}=-\frac{1}{\rho }\frac{\partial p}{\partial x}+\nu \frac{\partial^{2}u}{\partial z^{2}}-\frac{\sigma B_{0}^{2}u}{\rho }. \end{array}$$ (2)

The pressure gradient in the free stream can be obtained from Eq (2) as

$$\frac{1}{\rho }\frac{\partial p}{\partial x}=-\frac{\partial U}{\partial t}-U\frac{\partial U}{\partial x}-\frac{\sigma B_{0}^{2}U}{\rho },$$ (3)

so that Eq (2) becomes

$$\frac{\partial u}{\partial t}+u\frac{\partial u}{\partial x}+w\frac{\partial u}{\partial z}=\frac{\partial U}{\partial t}+U\frac{\partial U}{\partial x}+\nu \frac{\partial^{2}u}{\partial z^{2}}+\frac{\sigma B_{0}^{2}}{\rho }(U-u).$$ (4)

The appropriate boundary conditions with velocity partial slip are given by (see Jat and Rajotia [24]);

$$\begin{array}{ccc} u & = & u_{w}\left(x,t\right)+L_{1}\nu \frac{\partial u}{\partial z},\;v=cy/\left(1-\lambda t\right),\;w=0\;\text{at}\;z=0, \end{array}$$ (5)

$$\begin{array}{ccc} u & \to & U(x,t)=ax/(1-\lambda t),\;\;\text{as}\;\;z\to \infty . \end{array}$$ (6)

where *a*(> 0) is a constant. For the *u*-component boundary condition, we have assumed a velocity slip. This is proportional to local shear stress with slip factor *L*_1_ = *L*(1 − *λt*)^1/2^ where *L* is the initial velocity. Note that the essential slip factor *L*_1_ changes with time and has dimensions (velocity)^−1^. The velocity

$$\begin{array}{c} u_{w}\left(x,t\right)\;\left(=c\left(x+l\right)/\left(1-\lambda t\right)\right), \end{array}$$ (7)

is valid for time *t* < *λ* ^−1^.

We introduce the following similarity transformations to transform the govering equations

$$\begin{array}{c} u=\frac{\left[a\;x\;f^{\prime }\left(\eta \right)+c\;l\;h\left(\eta \right)\right]}{1-\lambda t},\;v=\frac{ayf^{\prime }\left(\eta \right)}{1-\lambda t},\;w=-2\sqrt{\frac{a\nu }{1-\lambda t}}\;f\left(\eta \right), \end{array}$$ (8)

where

$$\eta =z{\left(\frac{a}{\nu (1-\lambda t)}\right)}^{1/2},$$ (9)

and differentiation is with respect to *η*. Eqs (8) and (1) is identically satisfied. Substituting Eqs (8) and (9) in Eq (4) and equating the coefficients of *x* ^0^ and *x* ^1^, we obtain the coupled non-linear differential equations

$$\begin{array}{c} f^{\prime \prime \prime }+2ff^{\prime \prime }-f^{\prime^{2}}+1+M^{2}\left(1-f^{\prime }\right)-\beta [\frac{\eta }{2}f^{\prime \prime }+f^{\prime }-1]=0, \end{array}$$ (10)

$$\begin{array}{c} h^{\prime \prime }+2fh^{\prime }-hf^{\prime }-M^{2}h-\beta [\frac{\eta }{2}h^{\prime }+h]=0, \end{array}$$ (11)

where $B=B_{0}\sqrt{\left(1-\lambda t\right)}$. In Eqs (10) and (11), *β* = (*λ*/*a*) and *M* = (*σB* ^2^/*aρ*)^1/2^ are respectively the unsteadiness parameter and the magnetic parameter characterizing the strength of the imposed magnetic field.

The appropriate boundary conditions are obtained from Eqs (5) and (6) as

$$\begin{array}{ccc} f(0) & = & 0,\;f^{\prime }\left(0\right)=\alpha +\delta f^{\prime \prime }\left(0\right),\;f^{\prime }\left(\infty \right)=1, \end{array}$$ (12)

$$\begin{array}{ccc} h(0) & = & 1,\;h(\infty )=0,\; \end{array}$$ (13)

here *δ* = *L*(*aν*)^1/2^ is the dimensionless velocity slip parameter and *α* = (*c*/*a*) is the velocity ratio parameter. It is worth mentioning that the non-dimensional velocity slip parameter (*δ*) is always positive.

The non-dimensional velocity components is be introduced from the Eq (8) as

$$\begin{array}{ccc} u^{*} & = & u\sqrt{\frac{\left(1-\lambda t\right)}{a\nu }}=\xi f^{\prime }\left(\eta \right)+\alpha \;L\;h\left(\eta \right), \end{array}$$ (14)

$$\begin{array}{ccc} w^{*} & = & w\sqrt{\frac{\left(1-\lambda t\right)}{a\nu }}=-2f\left(\eta \right), \end{array}$$ (15)

where

$$\xi =x{\left(\frac{a}{\nu (1-\lambda t)}\right)}^{1/2}\;\text{and}\;L=l{\left(\frac{a}{\nu (1-\lambda t)}\right)}^{1/2}.$$ (16)

The dimensionless wall shear stress *τ* is then given by

$$\tau =\xi f^{\prime \prime }\left(0\right)+\alpha \;L\;h^{\prime }\left(0\right).$$ (17)

## 3 Heat transfer

The unsteady heat equation for a fluid with viscous and ohmic heating and variable thermal conductivity is given by (see Chiam [25])

$$\frac{\partial T}{\partial t}+\rho c_{p}(u\frac{\partial T}{\partial x}+w\frac{\partial T}{\partial z})=\frac{\partial }{\partial z}(\kappa \left(T\right)\frac{\partial T}{\partial z})+\mu {\left(\frac{\partial u}{\partial z}\right)}^{2}+\sigma B_{0}^{2}{\left(u-U\right)}^{2}-\frac{\partial q_{r}}{\partial z},$$ (18)

where*κ*(*T*), *c*_*p*_ and *q*_*r*_ are the temperature dependent thermal conductivity, the specific heat at constant pressure and the radiative heat flux of the fluid, respectively. The second term on the right hand side of Eq (18) represents the viscous dissipation in the flow; the third term stands for the dissipation of the magnetic energy in the form of Joule heating (Shercliff [26]) while the last term is due to the thermal radiation. Here, the temperature dependent thermal conductivity is written in the form (see Chiam [25])

$$\kappa \left(T\right)=\kappa_{\infty }[1+\frac{\epsilon }{\Delta T}],$$ (19)

where *κ*_∞_ denotes the conductivity of the fluid away from the surface, Δ*T* = *T*_*w*_ − *T*_∞_, *T*_∞_ and *T*_*w*_ are free stream temperature and the sheet temperature. *ϵ* is a small parameter. Substituting Eq (19) into Eq (18), gives

$$\frac{\partial T}{\partial t}+\rho c_{p}u\frac{\partial T}{\partial x}+(\rho c_{p}w-\frac{\kappa_{\infty }\epsilon }{\Delta T}\frac{\partial T}{\partial z})\frac{\partial T}{\partial z}=\kappa \left(T\right)\frac{\partial^{2}T}{\partial z^{2}}+\mu {\left(\frac{\partial u}{\partial z}\right)}^{2}+\sigma B_{0}^{2}{\left(u-U\right)}^{2}-\frac{\partial q_{r}}{\partial z},$$ (20)

where the radiation heat flux *q*_*r*_ is defined as

$$q_{r}=-\frac{4\sigma^{*}}{3k^{*}}\frac{\partial T^{4}}{\partial z},$$ (21)

where k* is the Rosseland mean absorption coefficient and σ* is denoted as the Stefan-Boltzmann constant. Here, Taylor series expansion is used to expand the temperature variation *T* ^4^ about *T*_∞_, and on neglecting higher order terms we obtain, $T^{4}≅4T_{\infty }^{3}T-3T_{\infty }^{4}$. Eq (20) becomes

$$\frac{\partial T}{\partial t}+\rho c_{p}u\frac{\partial T}{\partial x}+(\rho c_{p}w-\frac{\kappa_{\infty }\epsilon }{\Delta T}\frac{\partial T}{\partial z})\frac{\partial T}{\partial z}=(\kappa \left(T\right)+\frac{16\sigma^{*}}{3k^{*}})\frac{\partial^{2}T}{\partial z^{2}}+\mu {\left(\frac{\partial u}{\partial z}\right)}^{2}+\sigma B_{0}^{2}{\left(u-U\right)}^{2}.$$ (22)

The thermal boundary conditions may vary depending on the different types of heating processes under consideration. In this study, prescribed surface temperature and prescribed wall heat flux conditions are considered as two different examples of heating processes.

### 3.1 Case 1: Prescribed Surface Temperature (PST)

We assume that the prescribed wall temperature is a quadratic function in *x* (see Mahapatra and Nanday [17]) given by,

$$\begin{array}{c} T_{w}=T_{\infty }+A{\left(x/l_{1}\right)}^{2}{\left(1-\lambda t\right)}^{-3/2}\;\text{at}\;z=0, \end{array}$$ (23)

$$\begin{array}{c} T\to T_{\infty }\text{ as }z\to \infty , \end{array}$$ (24)

where *A* is a constant, *T*_*w*_ is the variable wall temperature and *l*_1_ is a reference length. The dimensionless temperature *θ* is defined as

$$\theta =\frac{T-T_{\infty }}{T_{w}-T_{\infty }}.$$ (25)

Substituting Eqs (23) and (25) into Eq (22), we get

$$\begin{array}{c} \left(1+\epsilon \theta +N_{r}\right)\theta^{\prime \prime }+\epsilon \theta^{\prime 2}+Pr[2f\theta^{\prime }-2\left(f^{\prime }+\alpha RLh\right)\theta +E_{c}{\left(f^{\prime \prime }+\alpha RLh^{\prime }\right)}^{2}\\ +E_{c}M^{2}{\left(f^{\prime }-1+\alpha RLh\right)}^{2}-\frac{\beta }{2}\left(\eta \theta^{\prime }+3\theta \right)]=0, \end{array}$$ (26)

where *N*_*r*_,*E*_*c*_ and *P*_*r*_ denote the radiation parameter, Eckert and Prandtl numbers, respectively. We defined these physical parameters as follows:

$$N_{r}=\frac{16\sigma^{*}T_{\infty }^{3}}{3\kappa_{\infty }k^{*}},\;P_{r}=\frac{\rho c_{p}}{\kappa_{\infty }},\;E_{c}=\frac{a^{2}l_{1}^{2}}{A_{0}c_{p}},\;R=\frac{1}{\xi }\;A=A_{0}/\sqrt{1-\lambda t},$$ (27)

with boundary conditions

θ(0)=1,θ(∞)=0. (28)

### 3.2 Case 2: Prescribed Wall Heat Flux (PHF)

The heat flux *q*_*w*_ at the surface is assumed to vary as the square of the distance as follows (see Mahapatra and Nanday [17]):

$$\begin{array}{c} -\kappa_{\infty }\frac{\partial T}{\partial z}=q_{w}=D{\left(x/l_{1}\right)}^{2}{\left(1-\lambda t\right)}^{-3/2}\;\text{at}\;z=0, \end{array}$$ (29)

$$\begin{array}{c} T\to T_{\infty }\;\text{as}\;z\to \infty , \end{array}$$ (30)

where *D* is a constant. Here we set

$$T-T_{\infty }=\frac{D}{\kappa_{\infty }}\sqrt{\frac{\nu }{a}}{\left(x/l_{1}\right)}^{2}{\left(1-\lambda t\right)}^{-3/2}g\left(\eta \right),$$ (31)

so that Eq (22), is transformed into the equation

$$\begin{array}{c} \left(1+\epsilon g+N_{r}\right)g^{\prime \prime }+\epsilon g^{\prime 2}+Pr[2fg^{\prime }-2\left(f^{\prime }+\alpha RLh\right)g+E_{c}{\left(f^{\prime \prime }+\alpha RLh^{\prime }\right)}^{2}\\ +E_{c}M^{2}{\left(f^{\prime }-1+\alpha RLh\right)}^{2}-\frac{\beta }{2}\left(\eta g^{\prime }+3g\right)]=0, \end{array}$$ (32)

with boundary conditions

$$g^{\prime }\left(0\right)=-1,\;g\left(\infty \right)=0,$$ (33)

where the Eckert number $E_{c}=\frac{\kappa_{\infty }a^{2}l_{1}^{2}\sqrt{a/\nu }}{D_{0}c_{p}}$ and *D* = *D*_0_/(1 − *λt*)^1/2^. Eq (32) has exactly the same form as Eq (26) but with a different first boundary condition.

## 4 Method of Solution

Eqs (10), (11) and (26) were solved using the successive relaxation method (SRM), Motsa [27]. The SRM is an iterative procedure that works in a similar fashion to the Gauss-Seidel method for algebraic equations. In this case the technique is used to linearize and decouple a system of differential equations. Further details of the rules of the SRM can be found in [28, 29].

The linear terms in each equation are evaluated at the current iteration level *r* + 1 and the non-linear terms are known from the previous iteration level *r*. The linearized form of Eqs (10), (11) and (26) are

$$f_{r+1}^{\prime \prime \prime }+a_{1,r}f_{r+1}^{\prime \prime }+a_{2,r}f_{r+1}^{\prime }=R_{1,r},$$ (34)

$$h_{r+1}^{\prime \prime }+b_{1,r}h_{r+1}^{\prime }+b_{2,r}h_{r+1}=R_{2,r},$$ (35)

$$\left(1+\epsilon \theta_{r}+Nr\right)\theta_{r+1}^{\prime \prime }+c_{r,1}\theta_{r+1}^{\prime }+c_{2,r}\theta_{r+1}=R_{3,r},$$ (36)

where

$$\begin{array}{l} a_{1,r}=2f_{r}-\frac{\beta \;\eta }{2},a_{2,r}=\beta +M^{2}-f_{r}^{\prime },\\ R_{1,r}=-\left[f_{r}^{\prime 2}+M^{2}+1+\beta \right],\\ b_{1,r}=2f_{r}-\frac{\beta \;\eta }{2},\;b_{2,r}=-\left[f_{r}^{\prime }+M^{2}+\beta \right],\;R_{2,r}=0,\\ c_{1,r}=2\epsilon \;\theta_{r}^{\prime }+2Pr\;f_{r}-\frac{Pr\beta \;\eta }{2},\;c_{2,r}=\epsilon \;\theta_{r}^{\prime \prime }-2Pr{\left(f_{r+1}^{\prime }+\alpha \;R\;L\;h\right)}^{2}-\frac{3Pr\;\beta }{2},\\ R_{3,r}=Pr\;E_{c}\left[{\left(f_{r}^{\prime \prime }+\alpha \;R\;L\;h_{r}^{\prime }\right)}^{2}+M^{2}{\left(f_{r}^{\prime }+\alpha \;R\;L\;h_{r}\right)}^{2}\right]-\epsilon \;\theta_{r}^{\prime \prime }\;\theta_{r}-\epsilon \;\theta_{r}^{\prime 2}. \end{array}$$

It must be noted that Eqs (34)–(36) are linear and decoupled and can thus be solved sequentially to obtain the quantities *f*(*η*), *h*(*η*) and *θ*(*η*). We opted in this study to use the Chebyshev spectral collocation method to discretize in *η* and finite differences with central differencing to discretize in *ξ*. Starting from initial guesses *f*_0_(*η*), *θ*_0_(*η*) and *ϕ*_0_(*η*), Eqs (34)–(36) were solved iteratively until the approximate solutions converged to within a certain prescribed tolerance level.

Similarly, for the PHF case, Eqs (10), (11) and (32) take the form

$$f_{r+1}^{\prime \prime \prime }+a_{1,r}f_{r+1}^{\prime \prime }+a_{2,r}f_{r+1}^{\prime }=R_{1,r},$$ (37)

$$h_{r+1}^{\prime \prime }+b_{1,r}h_{r+1}^{\prime }+b_{2,r}h_{r+1}=R_{2,r},$$ (38)

$$\left(1+\epsilon g_{r}+Nr\right)g_{r+1}^{\prime \prime }+c_{r,1}g_{r+1}^{\prime }+c_{2,r}g_{r+1}=R_{3,r},$$ (39)

where

$$\begin{array}{l} a_{1,r}=2f_{r}-\frac{\beta \;\eta }{2},a_{2,r}=\beta +M^{2}-f_{r}^{\prime },\\ R_{1,r}=-\left[f_{r}^{\prime 2}+M^{2}+1+\beta \right],\\ b_{1,r}=2f_{r}-\frac{\beta \;\eta }{2},\;b_{2,r}=-\left[f_{r}^{\prime }+M^{2}+\beta \right],\;R_{2,r}=0,\\ c_{1,r}=2\epsilon \;g_{r}^{\prime }+2Pr\;f_{r}-\frac{Pr\beta \;\eta }{2},\;c_{2,r}=\epsilon \;g_{r}^{\prime \prime }-2Pr{\left(f_{r+1}^{\prime }+\alpha \;R\;L\;h\right)}^{2}-\frac{3Pr\;\beta }{2},\\ R_{3,r}=Pr\;E_{c}\left[{\left(f_{r}^{\prime \prime }+\alpha \;R\;L\;h_{r}^{\prime }\right)}^{2}+M^{2}{\left(f_{r}^{\prime }+\alpha \;R\;L\;h_{r}\right)}^{2}\right]-\epsilon \;g_{r}^{\prime \prime }\;g_{r}-\epsilon \;g_{r}^{\prime 2}. \end{array}$$

## 5 Results and Discussion

The analysis of the results presented here relate to a decelerating shrinking sheet only (i.e., *β* ≤ 0) following Fang et al.[30], Rohini et al.[31] and Nandy et al.[32]. We have compared the local skin friction coefficients *f*′′(0) and *h*′(0) for various values of the parameter *α* with previously published data (Wang [15], Rahimpour et al. [33] and Mahapatra and Nandy [17]). The comparisons are shown in Table 1 where we observe an very good agreement to the results in the literature thus validating the current numerical results.

**Table 1:** Comparison table of the values of *f*′′(0) and *h*′(0) when *M* = 0 with recent literature.

	*f*′′(0)				*h*′(0)			
*α*	Wang [15]	Rahimpour et al. [33]	Mahapatra and Nanday [17]	Present Results	Wang [15]	Rahimpour et al. [33]	Mahapatra and Nanday [17]	Present Results
−0.95	0.9469	0.946815	0.946893	0.946897	0.26845	0.268450	0.268457	0.268458
−0.75	1.35284	1.352850	1.352841	1.352854	−0.22079	−0.220789	−0.220795	-0.220785
−0.50	1.49001	1.490004	1.352841	1.352852	−0.53237	−0.532371	−0.532374	-0.532379
−0.25	1.45664	1.456599	1.456641	1.456648	−0.75639	−0.756390	−0.756380	-0.756376
0.0	1.31193	1.311938	1.311942	1.311950	−0.93873	−0.938732	−0.938731	-0.938745
0.1	1.22911	1.229113	1.229111	1.229117	−1.00400	−1.004026	−1.004031	-1.004032
0.2	1.13374	1.133743	1.133750	1.133757	−1.06590	−1.065933	−1.065951	-1.065946
0.5	0.78032	0.780323	0.780327	0.780332	−1.23550	−1.235451	−1.235460	-1.235454
1.0	0	0	0	0	−1.47930	−1.479337	−1.479341	-1.479332
2.0	−2.13107	−2.131069	−2.131068	-2.131075	−1.88000	−1.879949	−1.879956	-1.879945
5.0	−11.8022	−11.802214	−11.802202	-11.802213	−2.76170	−2.761724	−2.761702	-2.76167

The phenomena of heat transfer is studied with respect to the numerical values of the physical parameters namely, (a) the wall temperature gradient ∣−*θ*′(0)∣ in the PST case and (b) the wall temperature ∣*g*(0)∣ in the PHF case. Tables 2 and 3 show that the wall temperature gradient ∣−*θ*′(0)∣ in the PST case and the wall temperature ∣*g*(0)∣ in the PHF case increases with increasing *M* when *α* and *ϵ* are fixed. The temperature gradient in the PST case and the wall temperature in the PHF case decrease with increases in the thermal conductivity parameter *ϵ*. We also observe that ∣−*θ*′(0)∣ and ∣ *g*(0) ∣ decrease as *α* increases in both the PST and the PHF cases.

**Table 2:** Wall temperature gradient ∣−*θ*′(0)∣ for the PST case taking *Pr* = 0.72, *R* = 1 *β* = −0.25, *L* = 1, *E*_*c*_ = 1 and *Nr* = 2.0.

*ϵ*	*α*	M = 0.0	M = 0.5	M = 1.0
0.0	-0.9	0.470476	0.515791	0.639041
	-0.3	0.469680	0.497224	0.575271
	-0.1	0.389930	0.402194	0.436688
0.1	-0.9	0.435015	0.477871	0.594412
	-0.3	0.434487	0.460699	0.535030
	-0.1	0.358828	0.370508	0.403393
0.2	-0.9	0.403350	0.443995	0.554504
	-0.3	0.403116	0.428120	0.499084
	-0.1	0.331167	0.342317	0.373743

**Table 3:** Wall temperature gradient ∣*g*(0)∣ for the PHF case taking *Pr* = 0.72, *R* = 1 *β* = −0.25, *L* = 1, *E*_*c*_ = 1 and *Nr* = 2.0.

*ϵ*	*α*	M = 0.0	M = 0.5	M = 1.0
0.0	-0.9	0.649572	0.681891	0.764965
	-0.3	0.593890	0.618274	0.686943
	-0.1	0.505823	0.517244	0.549463
0.1	-0.9	0.616233	0.648325	0.731028
	-0.3	0.555240	0.579129	0.646413
	-0.1	0.467061	0.478199	0.509608
0.2	-0.9	0.585066	0.616894	0.699120
	-0.3	0.519071	0.542448	0.608305
	-0.1	0.430932	0.441781	0.472366

**Fig 2:**
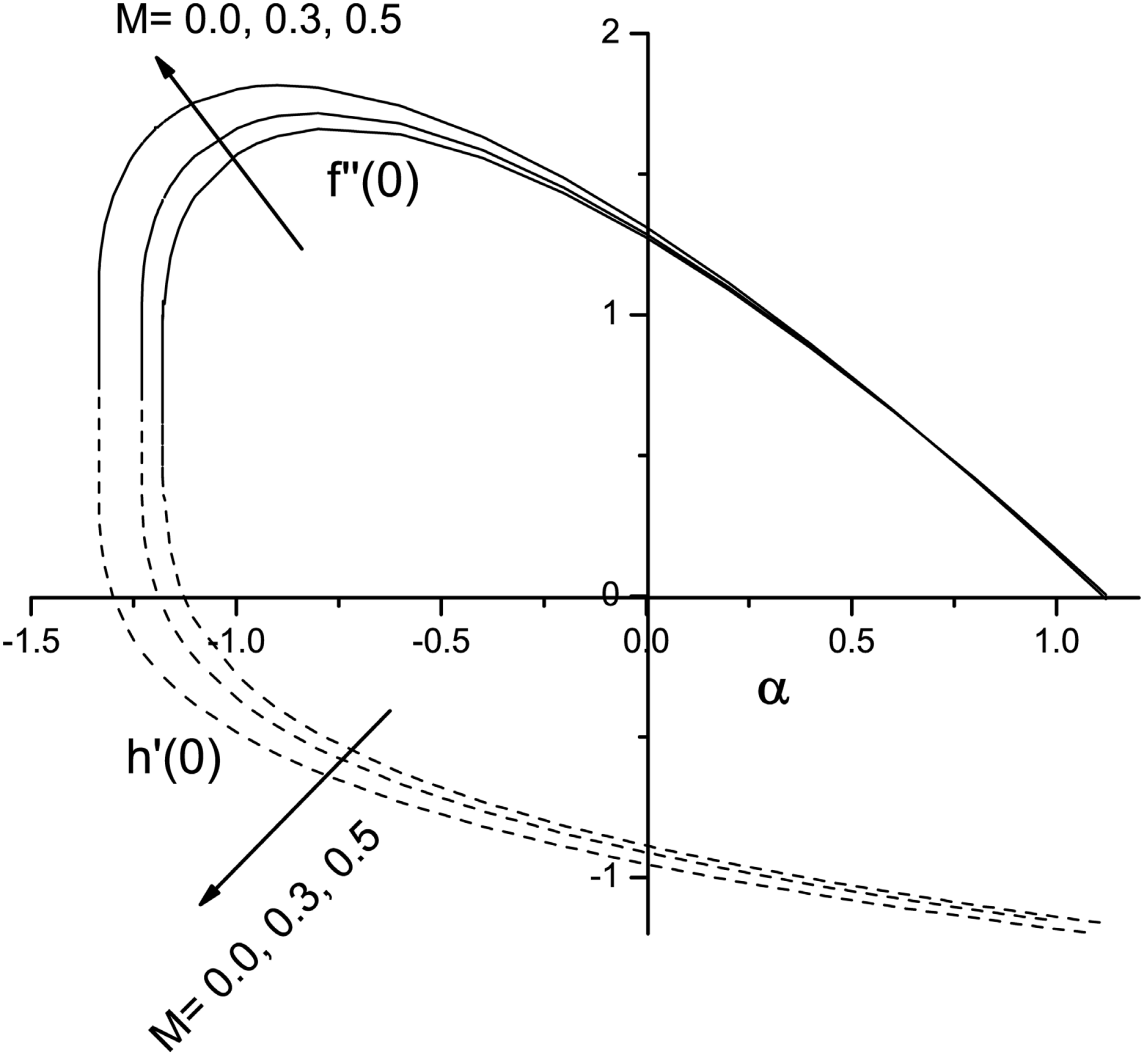
Initial values *F*′′(0) and *h*′(0) versus *α* and *M*.

Fig 2 depicts the variation of the skin friction coefficients *f*′′(0) and *h*′(0) with *α* < 0 (shrinking sheet) and *α* > 0 (stretching sheet) for different values of the magnetic parameter *M*. Here solid and dashed lines represent the trajectories of *f*′′(0) and *h*′(0), respectively. Our numerical results reveal that without a magnet (i.e., *M* = 0), Eqs (14) and (15) have unique solutions when *α* ≥ −1 and no similarity solution exists for *α* < −1. It is observed that the similarity solution exists up to a critical value *α* = *α*_*c*_(< 0), (say) beyond which a solution based on the boundary layer approximations does not exist as the boundary layer separates from the surface. From a physical point of view, a steady solution is not possible unless additional fluid from the stagnation-point is added to the free stream. A steady solution is possible only when ratio of the free stream velocity and shrinking velocity is less than a certain numerical value which again depends on the magnetic field parameter (*M*). The results show that when *M* increases, the range of *α* where similarity solutions exist gradually increases. When *α* = 1, we find that *f*′′(0) = 0 because *f*(*η*) = *η* is the solution of Eq (14) subject to the boundary conditions Eq (16). The results show that when *f*′′(0) ≥ 0, for a given value of *α*, *f*′′(0) increases with *M*. For a shrinking surface, the *h*′(0) orbits intersect the *α*-axis but this is not the case for flow over a stretching sheet. For a given value of *M*, the size of *h*′(0) decreases with increases in ∣*α*∣. Also, for any given *α*, ∣*h*′(0)∣ increases with *M*.

**Fig 3:**
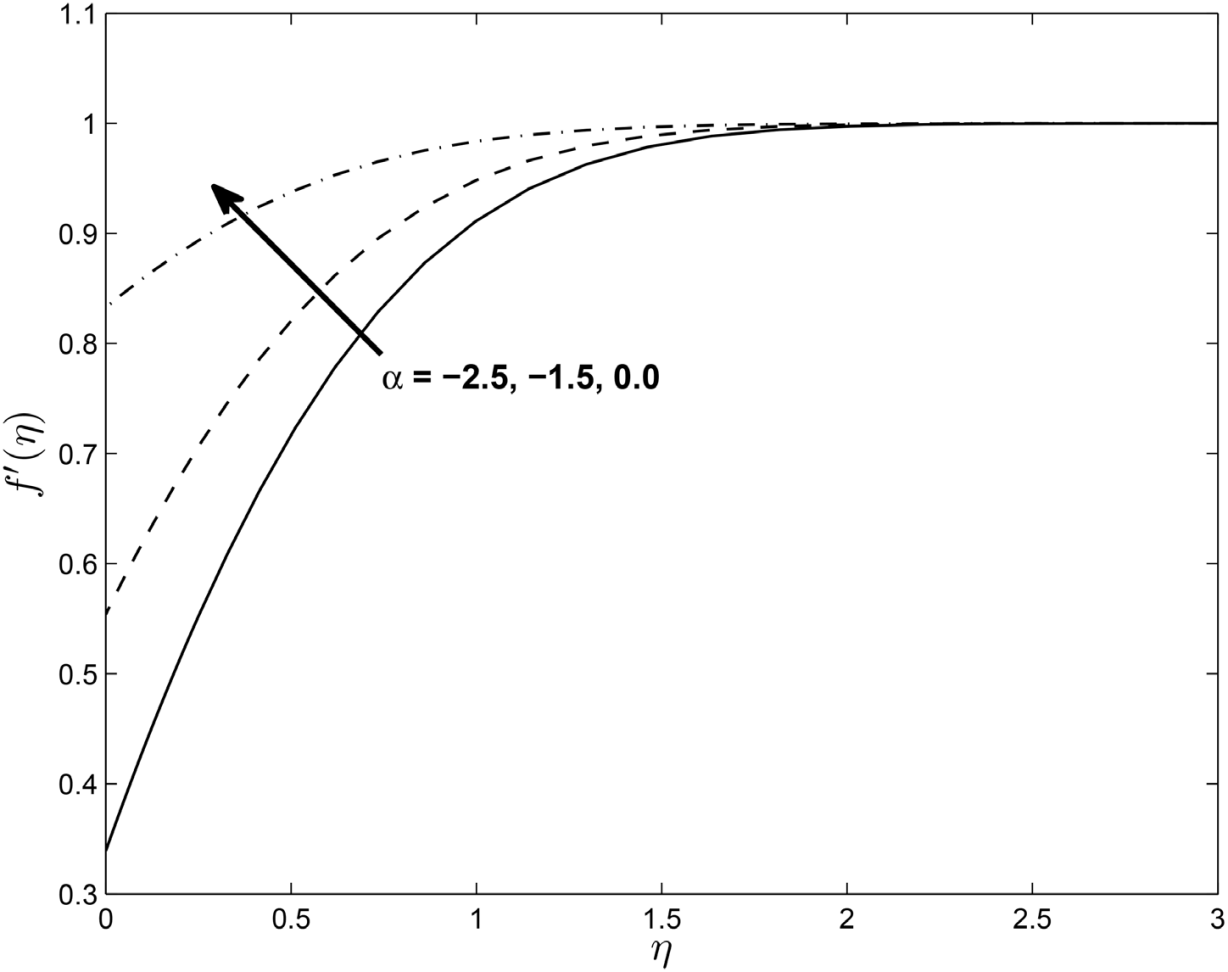
Effect of *α* on velocity profiles *f*′(*η*) for *M* = 0.1, *δ* = 0.2, *β* = −0.25, *Pr* = 0.72, *Nr* = 0.2, *Ec* = 1.0, *R* = 1.0 and *ϵ* = 0.5.

**Fig 4:**
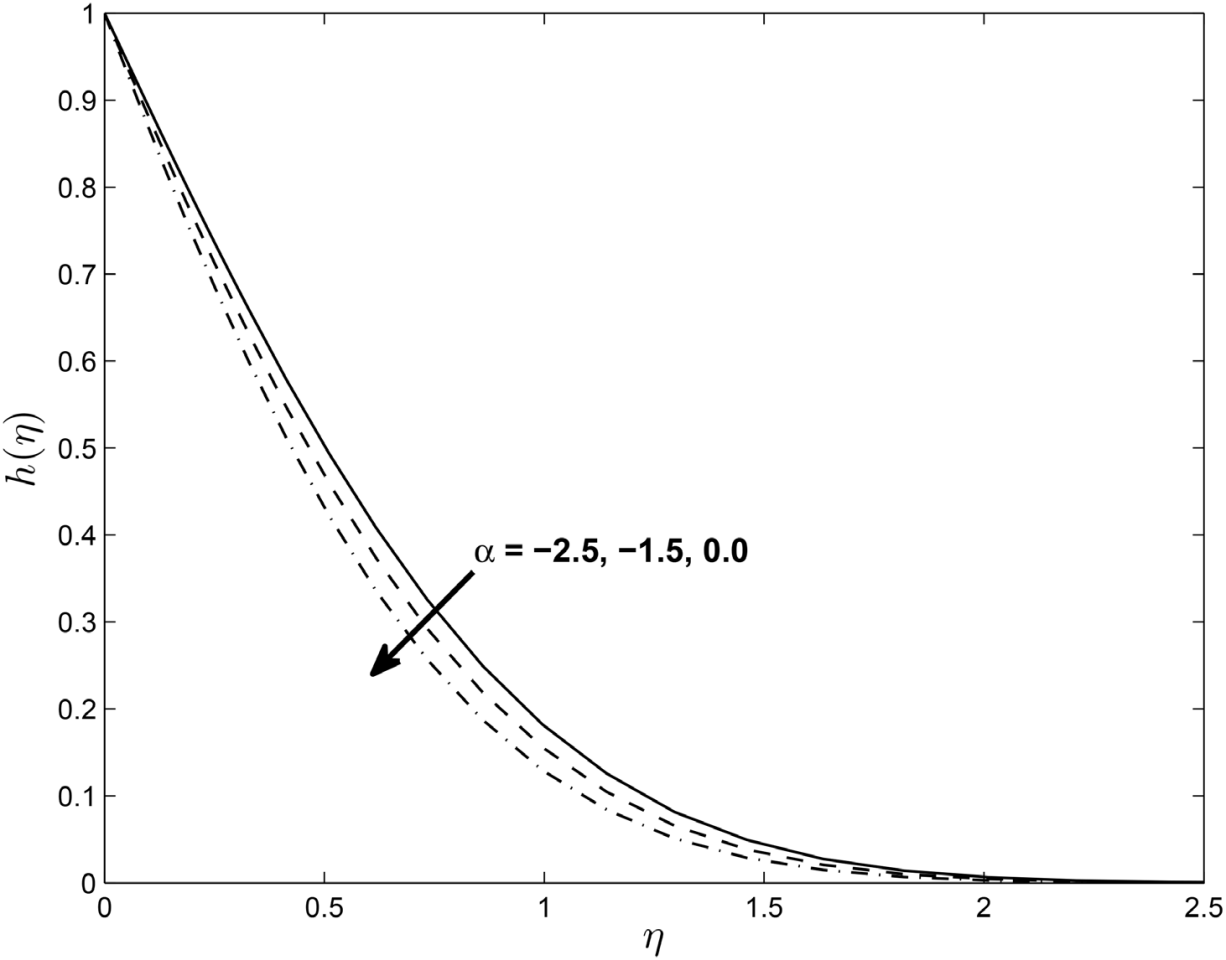
Effect of *α* on velocity profiles *h*(*η*) for *M* = 0.1, *δ* = 0.2, *β* = −0.25, *Pr* = 0.72, *Nr* = 0.2, *Ec* = 1.0, *R* = 1.0 and *ϵ* = 0.5.

We note that for a stretching sheet *α* is positive and for a shrinking sheet *α* is negative while *α* = 0 represents Hiemenz flow. Figs 3 and 4 show the effect of *α* on the vertical velocity components *f*′(*η*) and *h*(*η*). We observe that *f*′(*η*) increases with increases in *α* while the value of *h*(*η*) decreases with increases in the values of *α*.

**Fig 5:**
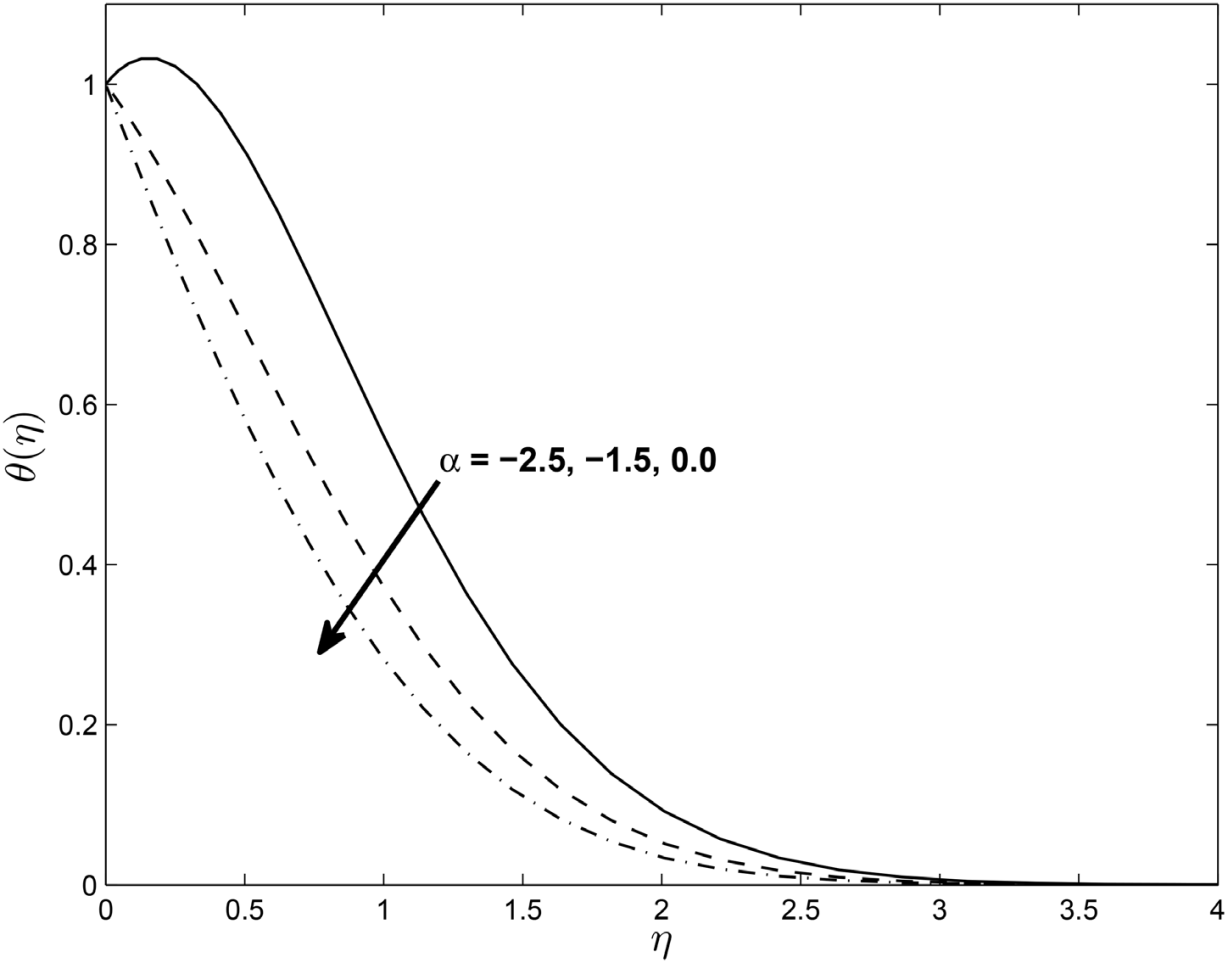
Effect of *α* on temperature profile for *M* = 0.1, *δ* = 0.2, *β* = −0.25, *Pr* = 0.72, *Nr* = 0.2, *Ec* = 1.0, *R* = 1.0, *L* = 1.0 and *ϵ* = 0.5.

**Fig 6:**
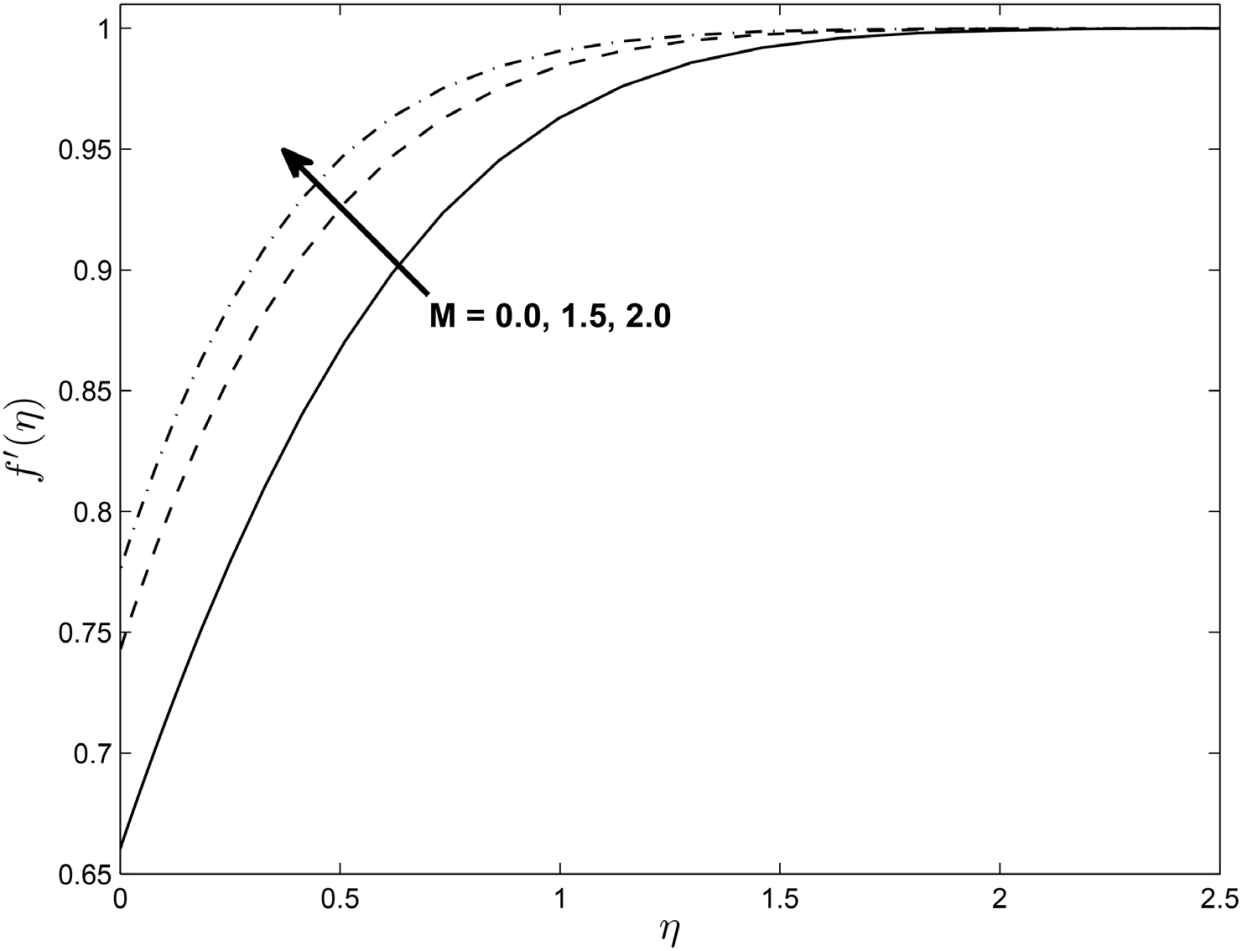
Effect of magnetic parameter *M* on velocity profiles *f*′(*η*) for *δ* = 0.2, *β* = −0.25, *α* = −0.95, *Pr* = 0.72, *Nr* = 0.2, *Ec* = 1.0, *R* = 1.0, *L* = 1.0 and *ϵ* = 0.5.

**Fig 7:**
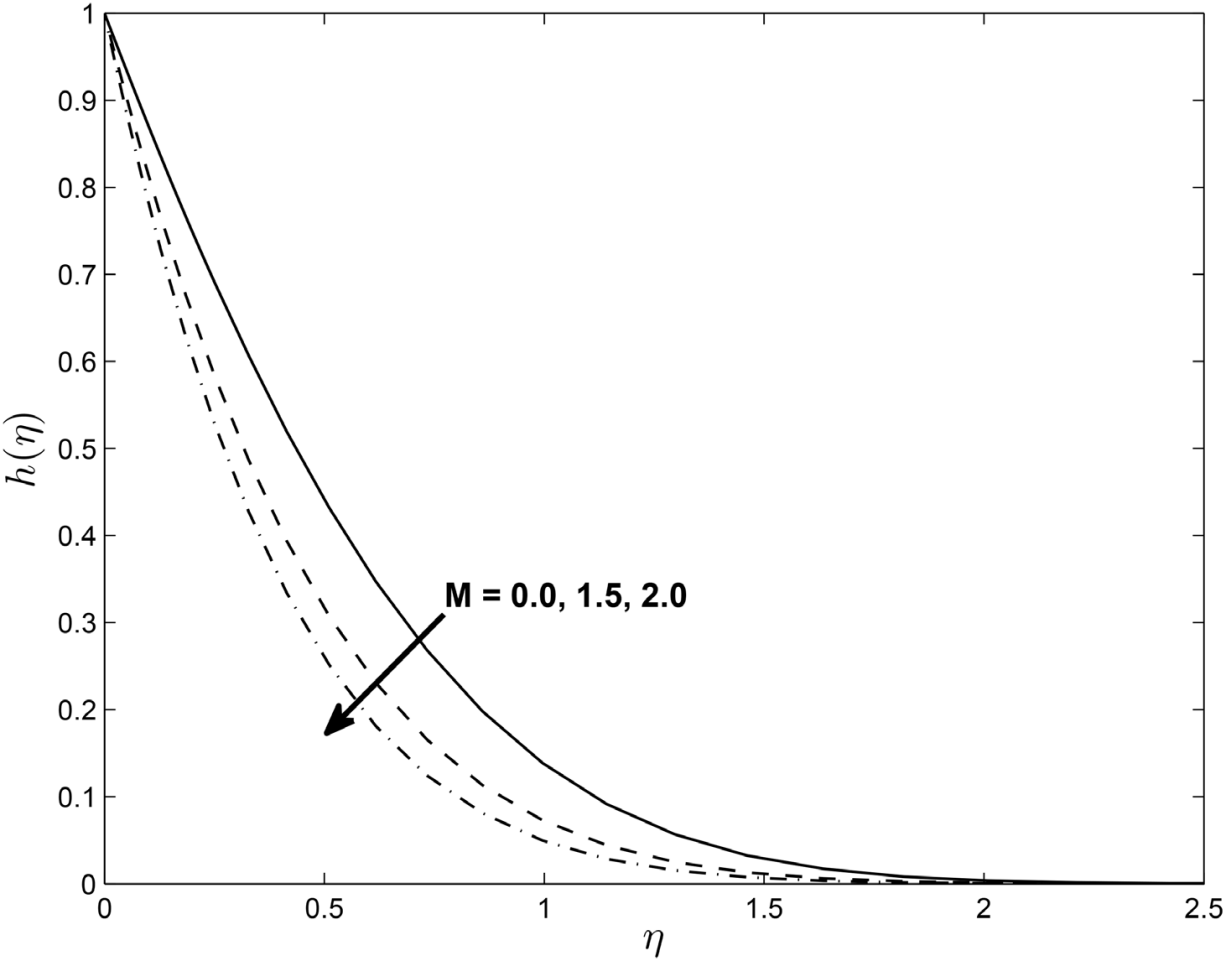
Effect of magnetic parameter *M* on velocity profiles *h*(*η*) for *δ* = 0.2, *β* = −0.25, *α* = −0.95, *Pr* = 0.72, *Nr* = 0.2, *Ec* = 1.0, *R* = 1.0, *L* = 1.0 and *ϵ* = 0.5.

Fig 5 displays the effect of *α* on the temperature profiles *θ*(*η*) (for PST case). Here the temperature profiles decrease with an increase in *α*. Figs 6 and 7 show the effect of *M* on *f*′(*η*) and non-alignment variable *h*(*η*) with respect to *η*, respectively. It is clear that *f*′(*η*) increases with increasing values of the magnetic parameter *M* and *h*(*η*) decreases with *M*. We can conclude from the above results is that for shrinking sheet, the effect of non-alignment becomes less pronounced with increasing *M*.

**Fig 8:**
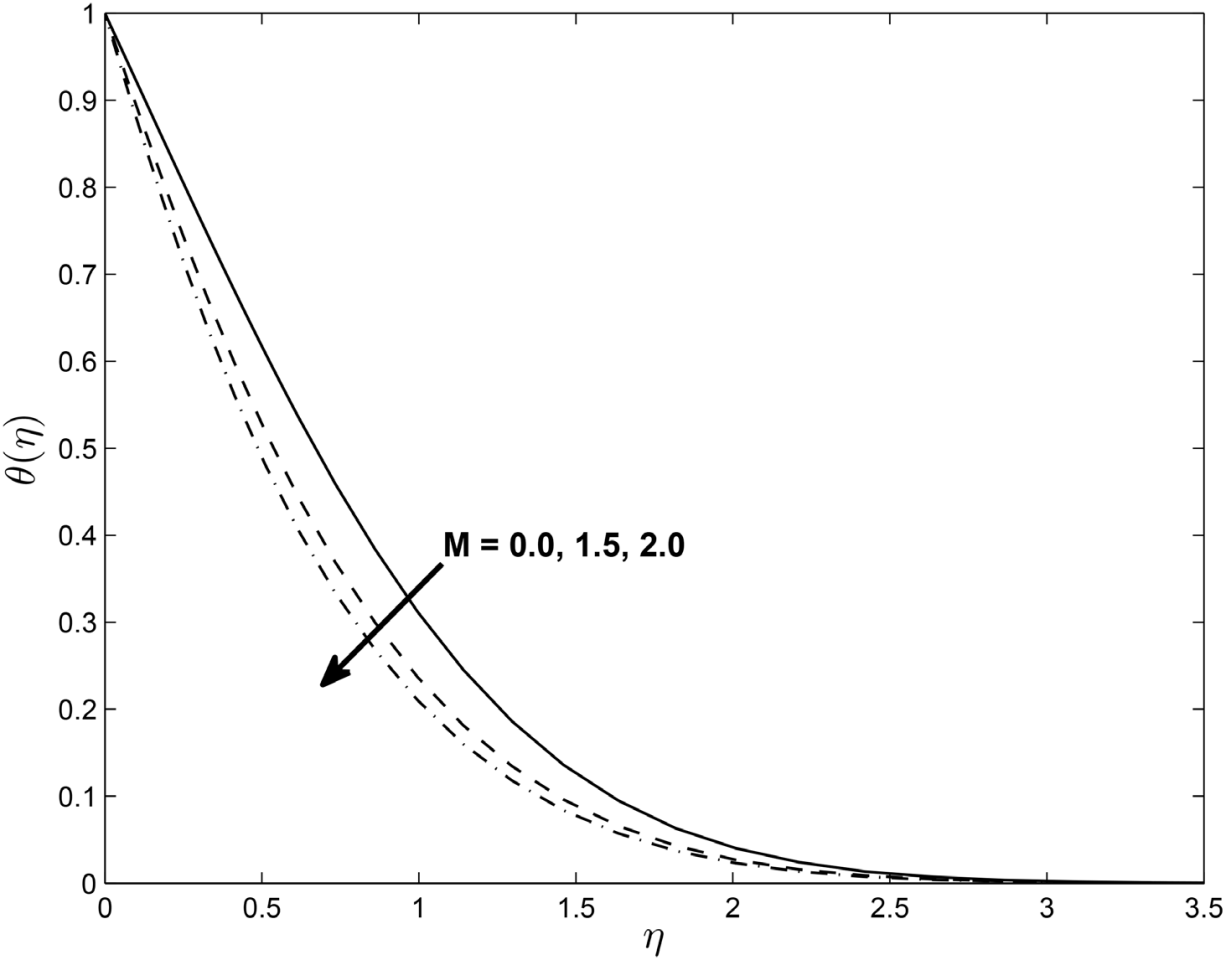
Effects of magnetic parameter *M* on temperature profiles (PST case) for *δ* = 0.2, *β* = −0.25, *α* = −0.95, *Pr* = 0.72, *Nr* = 0.2, *Ec* = 1.0, *R* = 1.0 and *L* = 1.0.

**Fig 9:**
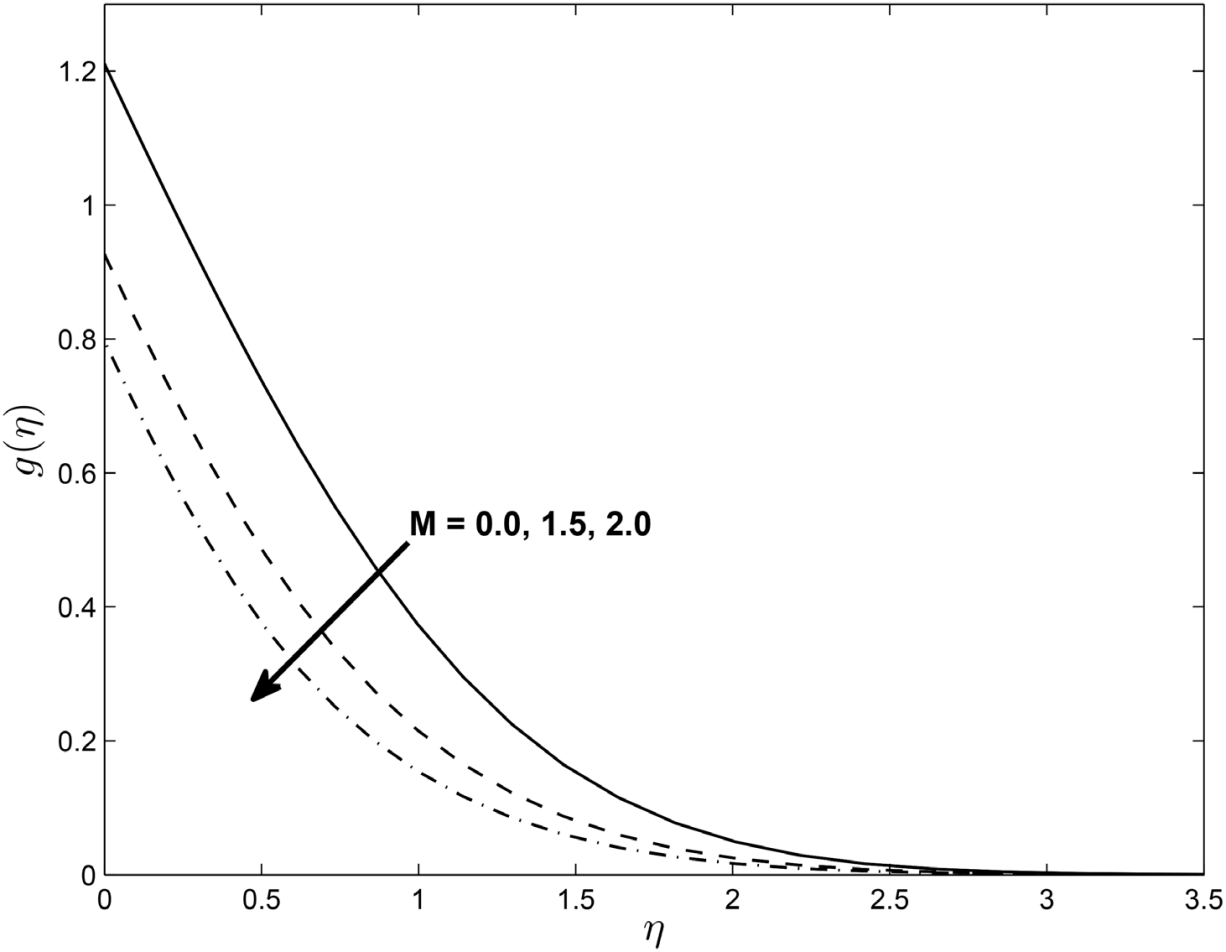
Effects of magnetic parameter *M* on temperature profiles (PHF case) for *δ* = 0.2, *β* = −0.25, *α* = −0.95, *Pr* = 0.72, *Nr* = 0.2, *Ec* = 1.0, *R* = 1.0 and *L* = 1.0.

**Fig 10:**
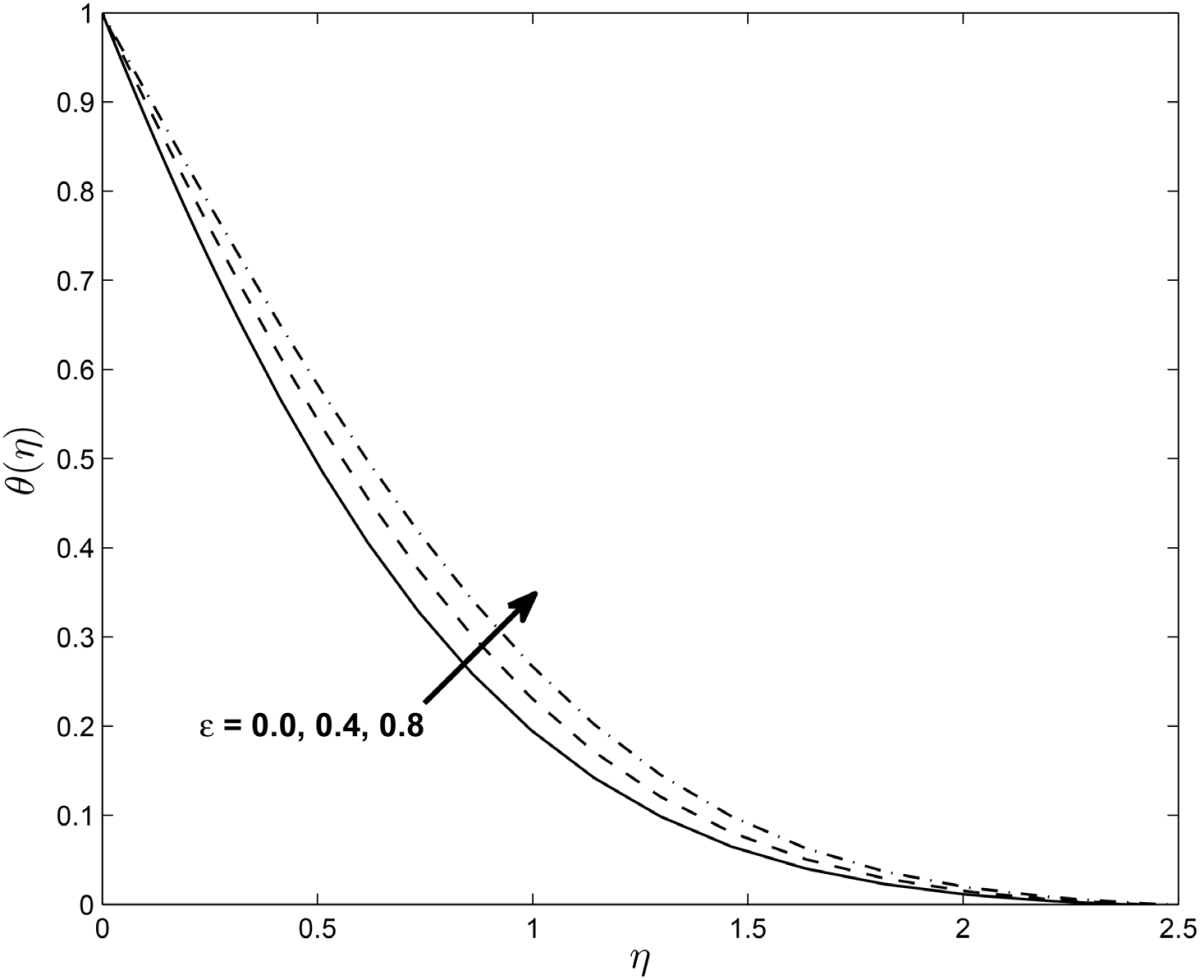
Effects of *ϵ* on temperature profiles (PST case) for *δ* = 0.2, *β* = −0.25, *α* = −0.95, *Pr* = 0.72, *Nr* = 0.2, *Ec* = 1.0, *R* = 1.0 and *L* = 1.0.

**Fig 11:**
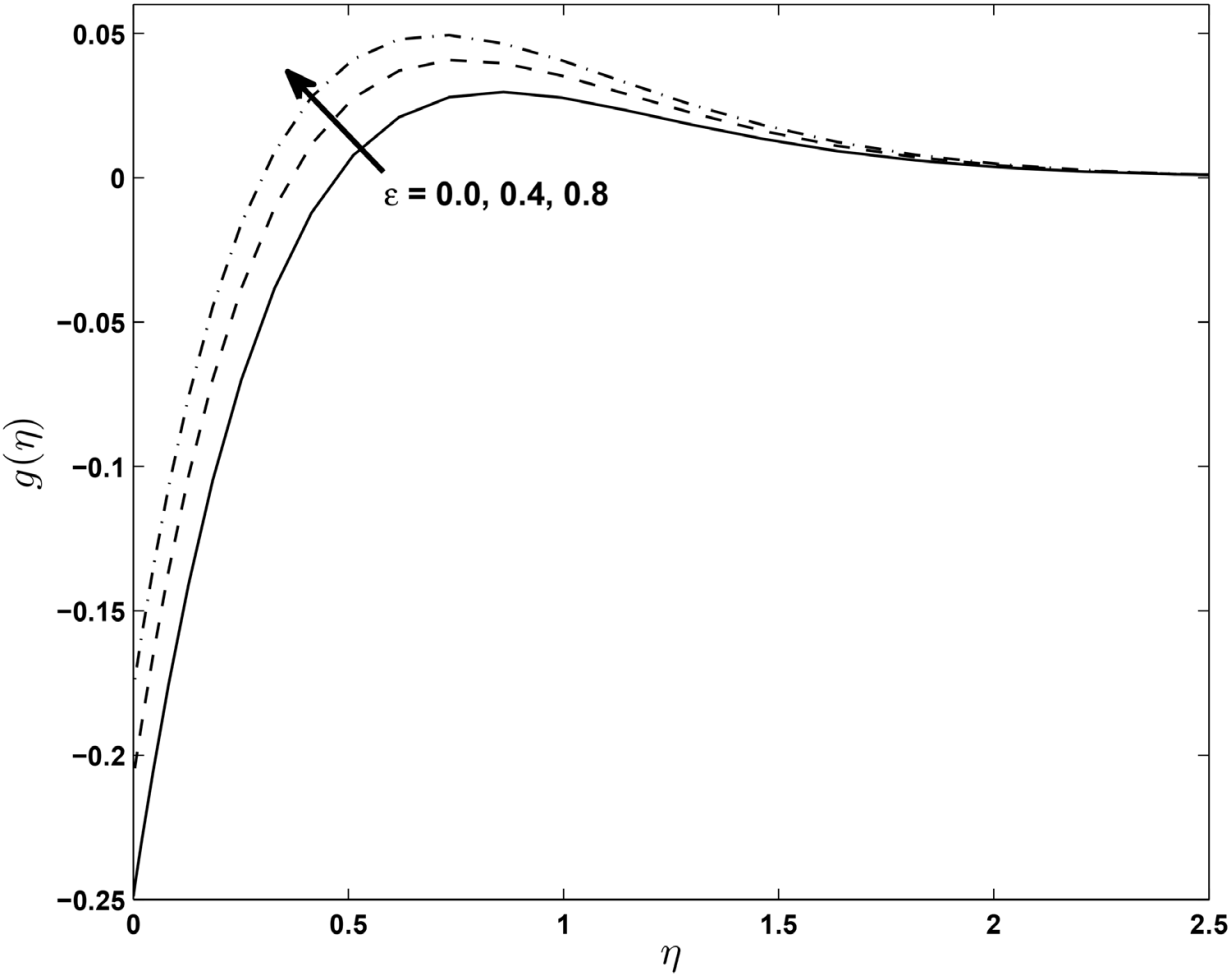
Effects of *ϵ* on temperature profiles (PHF case) for *δ* = 0.2, *β* = −0.25, *α* = −0.95, *Pr* = 0.72, *Nr* = 0.2, *Ec* = 1.0, *R* = 1.0 and *L* = 1.0.

Figs 8 and 9 show that the temperature profiles decrease monotonically with an increase in the magnetic parameter in both the PST and the PHF cases, respectively. The extent of the reverse circular flow above the sheet decreases with increases in *M*. This is a consequence of the fact that the temperature field given by Eq (18) is influenced by the advection of the fluid velocity above the sheet. Figs 10 and 11 exhibit the temperature profiles for different values of thermal conductivity parameter *ϵ* where the other parameters are fixed for both the PST and PHF cases, respectively. The temperature profiles increase with an increase in the thermal conductivity parameter due to increases in the thermal boundary layer thickness in both the PST and PHF cases.

**Fig 12:**
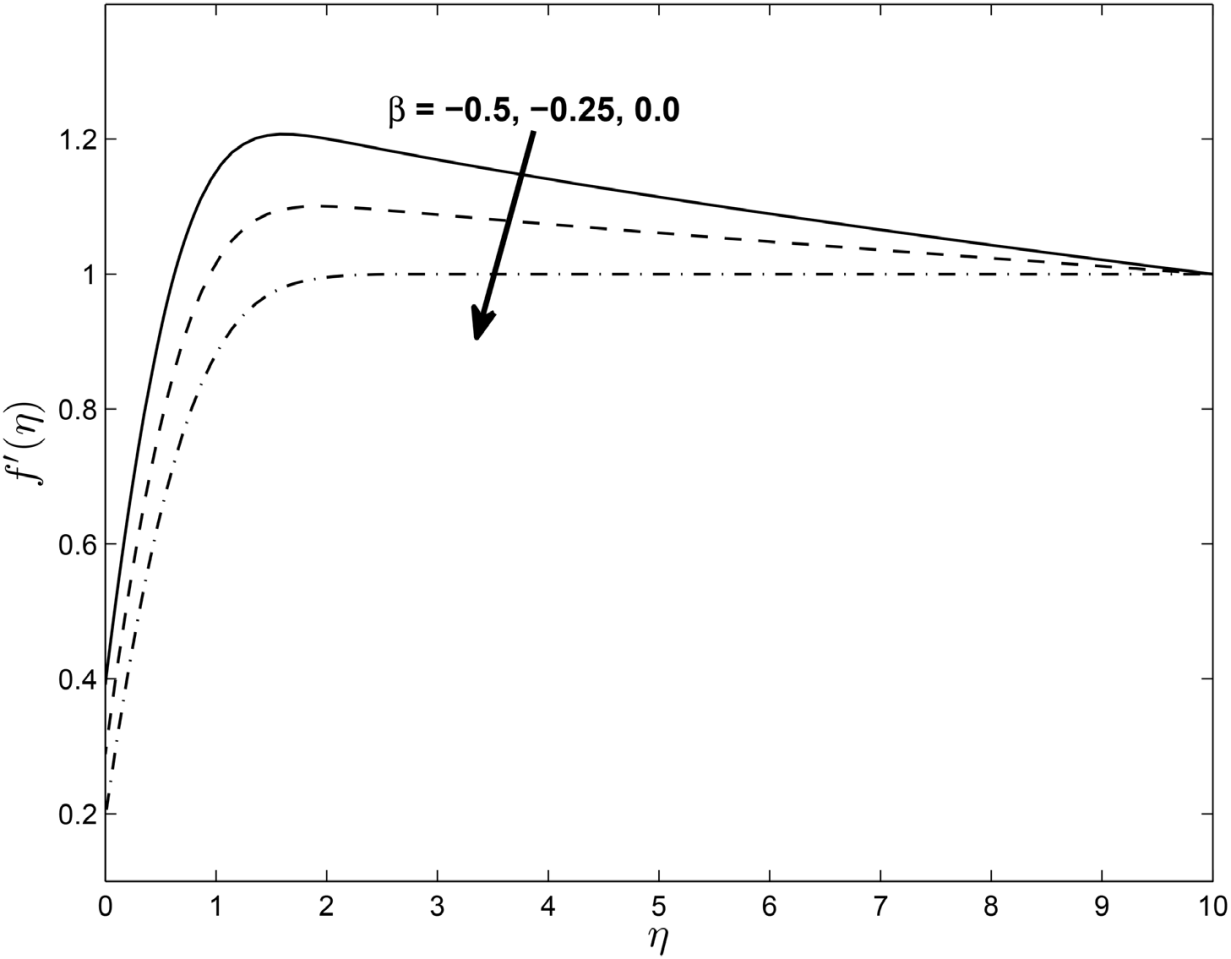
Effect of *β* on velocity profiles *f*′(*η*) for *M* = 0.1, *δ* = 0.2, *α* = −0.95, *Pr* = 0.72, *Nr* = 0.2, *Ec* = 1.0, *R* = 1.0, *L* = 1.0 and *ϵ* = 0.5.

**Fig 13:**
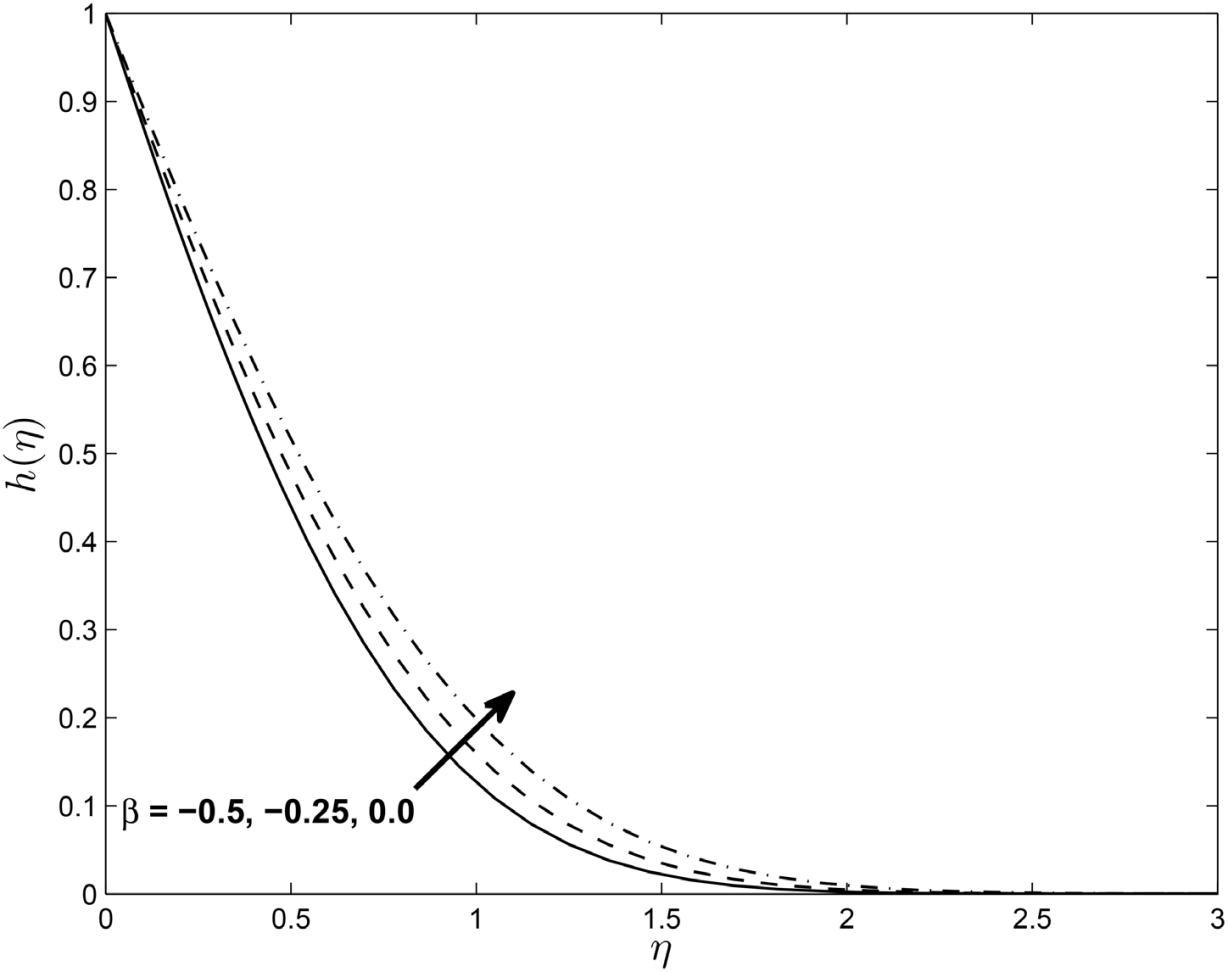
Effect of *β* on velocity profiles *h*(*η*) for *M* = 0.1, *δ* = 0.2, *α* = −0.95, *Pr* = 0.72, *Nr* = 0.2, *Ec* = 1.0, *R* = 1.0, *L* = 1.0 and *ϵ* = 0.5.

Figs 12 and 13 depict the horizontal velocity profiles *f*′(*η*) and *h*(*η*) for different values of the unsteadiness parameter *β* in the presence of slip at the boundary, respectively. The velocity *f*′(*η*) decreases with an increase in the unsteadiness parameter *β* and this implies an accompanying reduction in the thickness of the momentum boundary layer while the opposite trend is observed with *h*(*η*). We observe that as *β* increases, the axial boundary layer velocity decreases. In the vicinity of the sheet, the axial fluid velocity decreases while the trend is reversed in the free stream. The parameter *β* has the effect of reducing the momentum boundary layer thickness for *f*′(*η*) while enhancing the boundary layer thickness of *h*(*η*)

**Fig 14:**
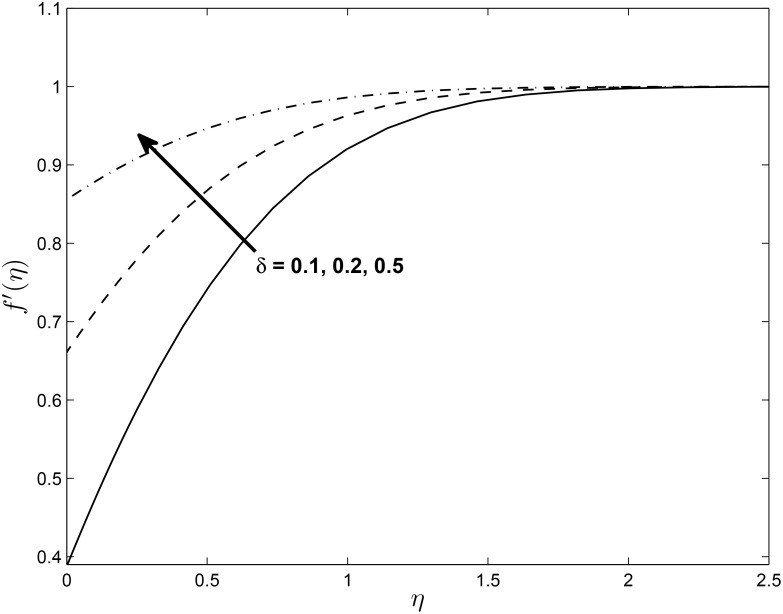
Effect of *δ* on velocity profiles *f*′(*η*) for *M* = 0.1, *β* = −0.25, *α* = −0.95, *Pr* = 0.72, *Nr* = 0.2, *Ec* = 1.0, *R* = 1.0, *L* = 1.0 and *ϵ* = 0.5.

**Fig 15:**
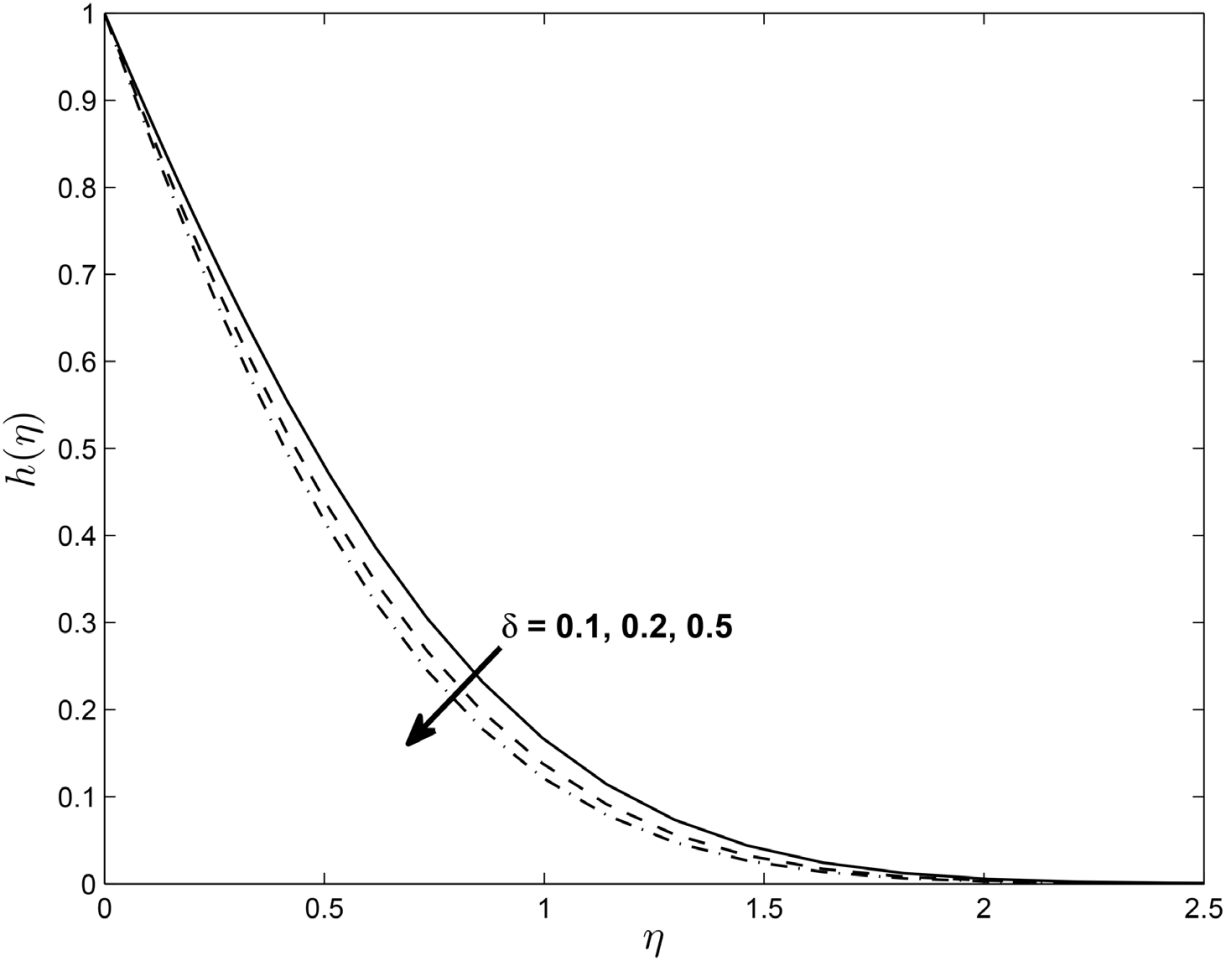
Effect of *δ* on velocity profiles *h*(*η*) for *M* = 0.1, *β* = −0.25, *α* = −0.95, *Pr* = 0.72, *Nr* = 0.2, *Ec* = 1.0, *R* = 1.0, *L* = 1.0 and *ϵ* = 0.5.

**Fig 16:**
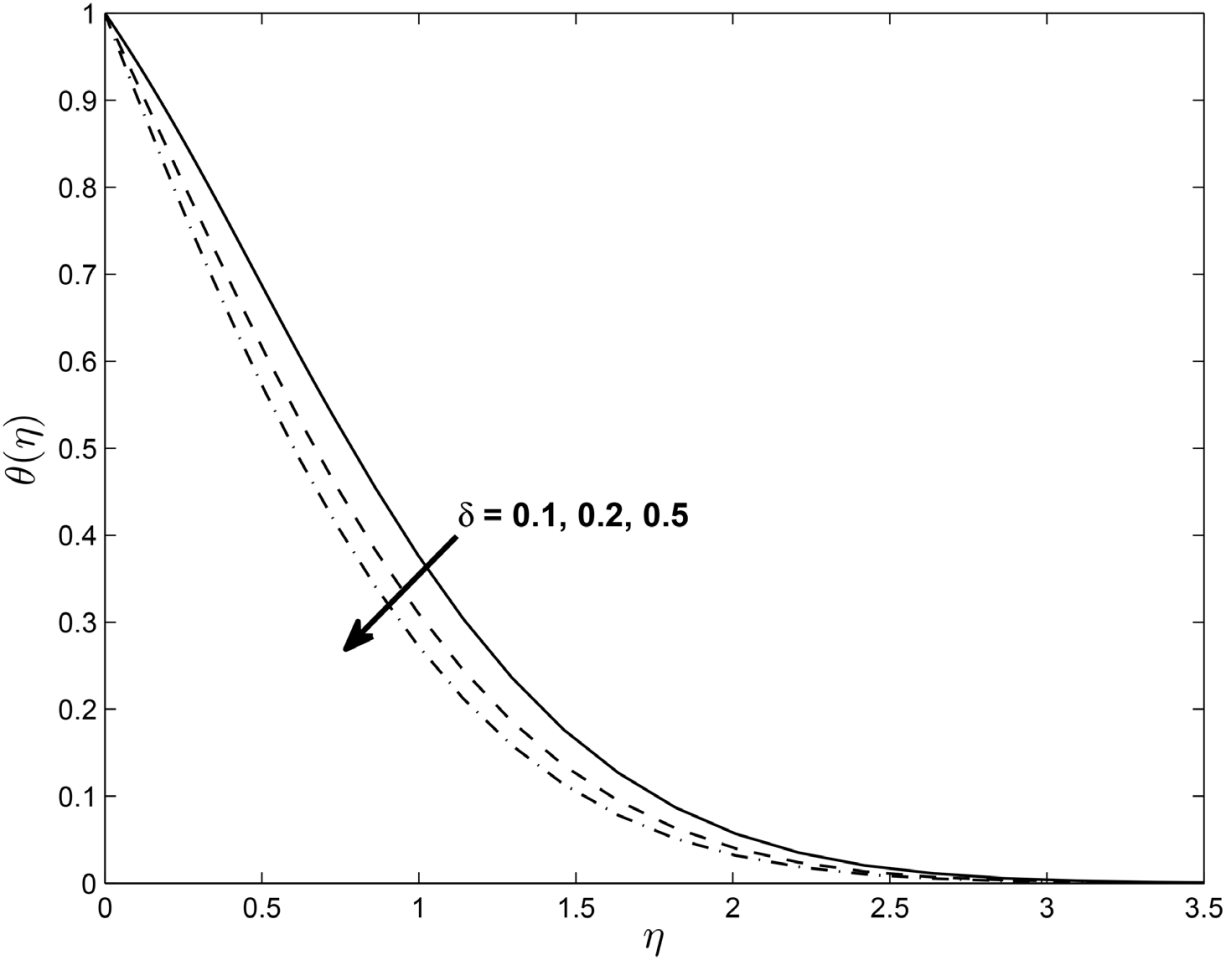
Effect of *δ* on temperature profile (PST case) for *M* = 0.1, *β* = −0.25, *α* = −0.95, *Pr* = 0.72, *Nr* = 0.2, *Ec* = 1.0, *R* = 1.0, *L* = 1.0 and *ϵ* = 0.5.

Figs 14, 15 and 16 show the effect of *δ* on the velocity components *f*′(*η*), *h*(*η*) and temperature profile *θ*(*η*) (for PST case), respectively. It is interesting to note that the velocity profile *f*′(*η*) increases with increase in values of *δ* while *h*(*η*) decreases with the increase in the values of *δ*. The figure also reveals that the temperature profile *θ*(*η*) decreases with the increase in the values of *δ*. This may be explained in the following way; with slip, there is a difference between the flow velocity near the sheet and the shrinking velocity at the surface. As *δ* increases the slip velocity increases leading to a decrease in the fluid velocity for *h*(*η*). But the opposite trend is observed for *f*′(*η*) because momentum boundary layer become thinner due to increasing value of *δ*. Fig 16 illustrates the fact that the temperature at any given point increases when the slip velocity *δ* increases.

**Fig 17:**
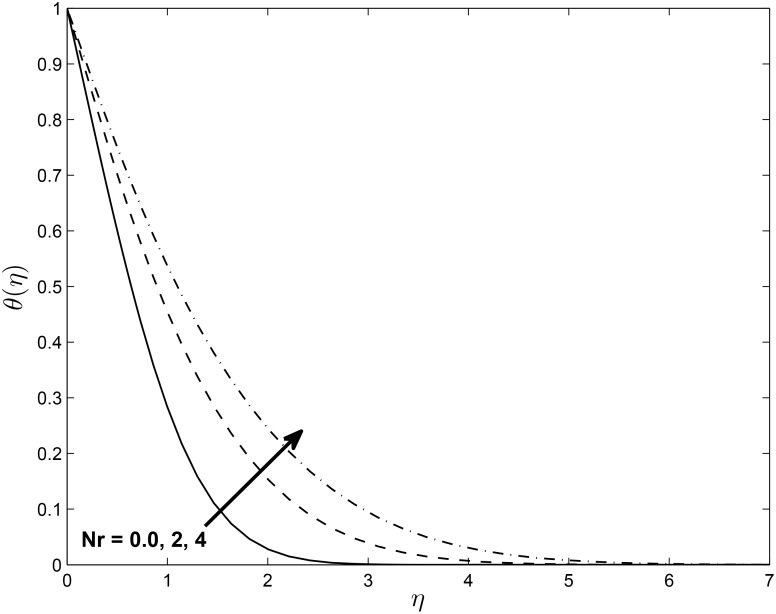
Effects of thermal radiation parameter *Nr* on temperature profiles *θ*(*η*) for *δ* = 0.2, *Pr* = 0.72, *β* = −0.25, *Ec* = 1.0, *α* = −0.95, *M* = 0.1, *R* = 1.0 and *L* = 1.0.

**Fig 18:**
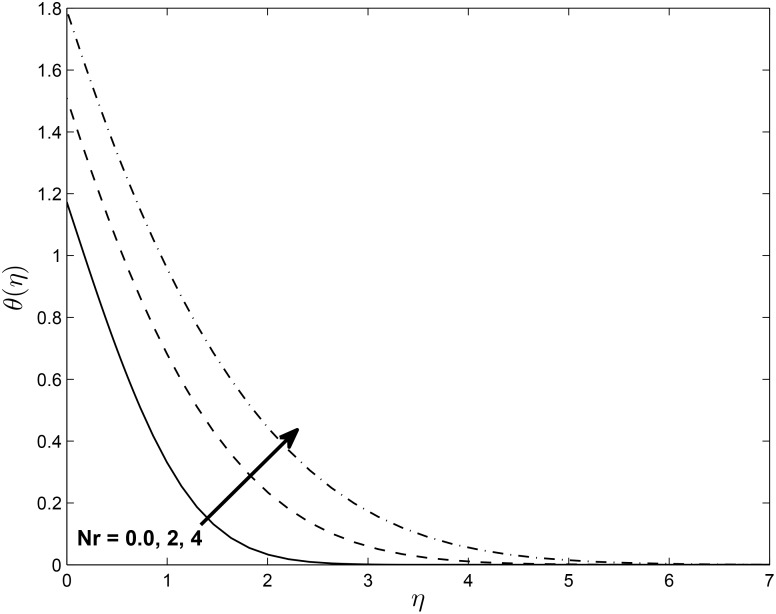
Effects of thermal radiation parameter *Nr* on temperature profiles *g*(*η*) for *δ* = 0.2, *Pr* = 0.72, *β* = −0.25, *Ec* = 1.0, *α* = −0.95, *M* = 0.1, *R* = 1.0 and *L* = 1.0.

Figs 17 and 18 depict the effect of *Nr* on the temperature profile in PST and PHF cases with keeping other parameters fixed, respectively. The temperature profile in two cases increase with increasing in values of *Nr*, which in turn increases the thermal boundary layer thickness for both PST and PHF cases. This may due to the fact that increases in the value of *N*_*r*_ causes an increase in the interaction with the thermal boundary layer.

**Fig 19:**
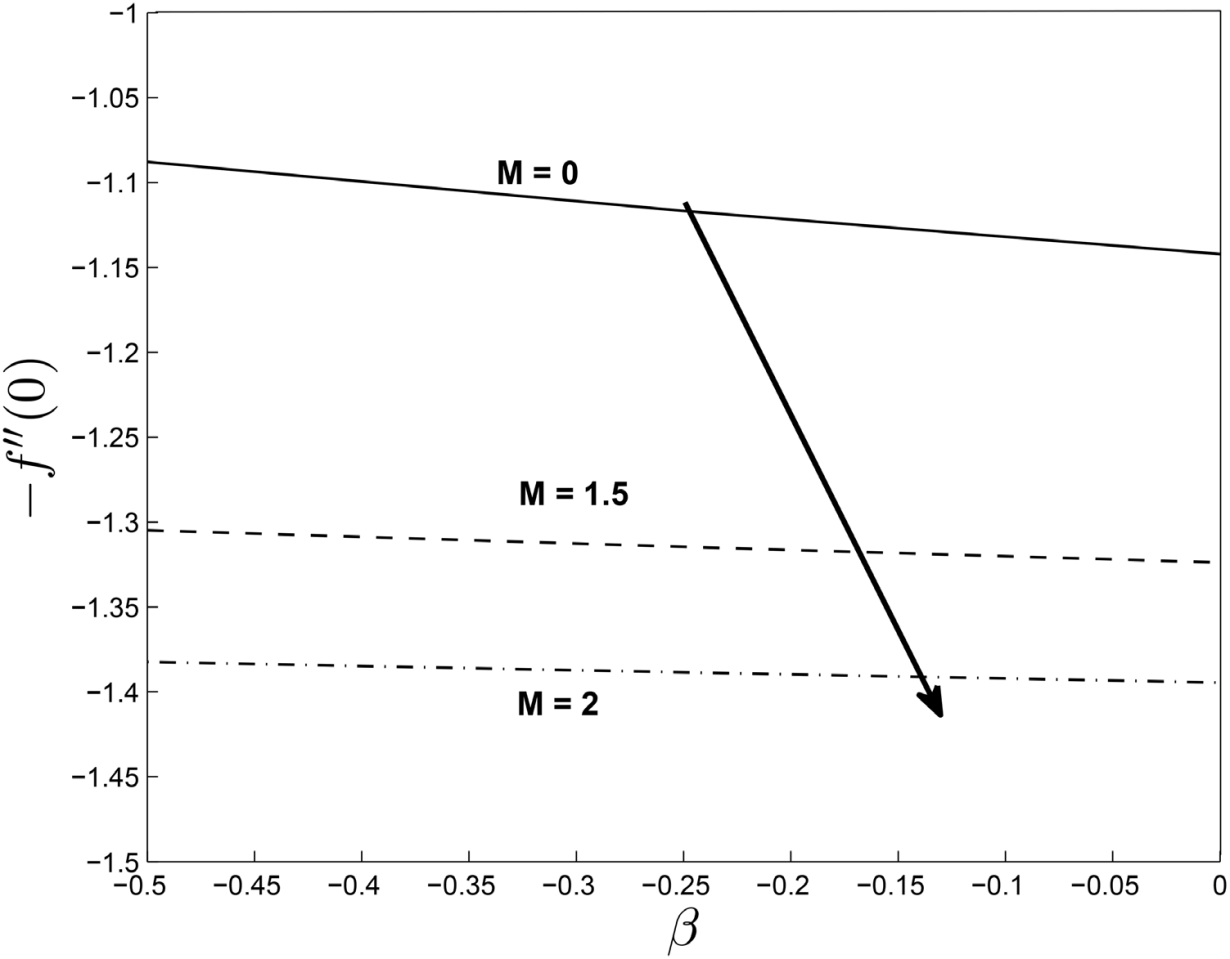
Effect of *M* on Skin friction coefficients for *α* = −0.95, *Pr* = 0.72, *Nr* = 0.2, *Ec* = 1.0, *R* = 1.0, *L* = 1.0 and *ϵ* = 0.5.

**Fig 20:**
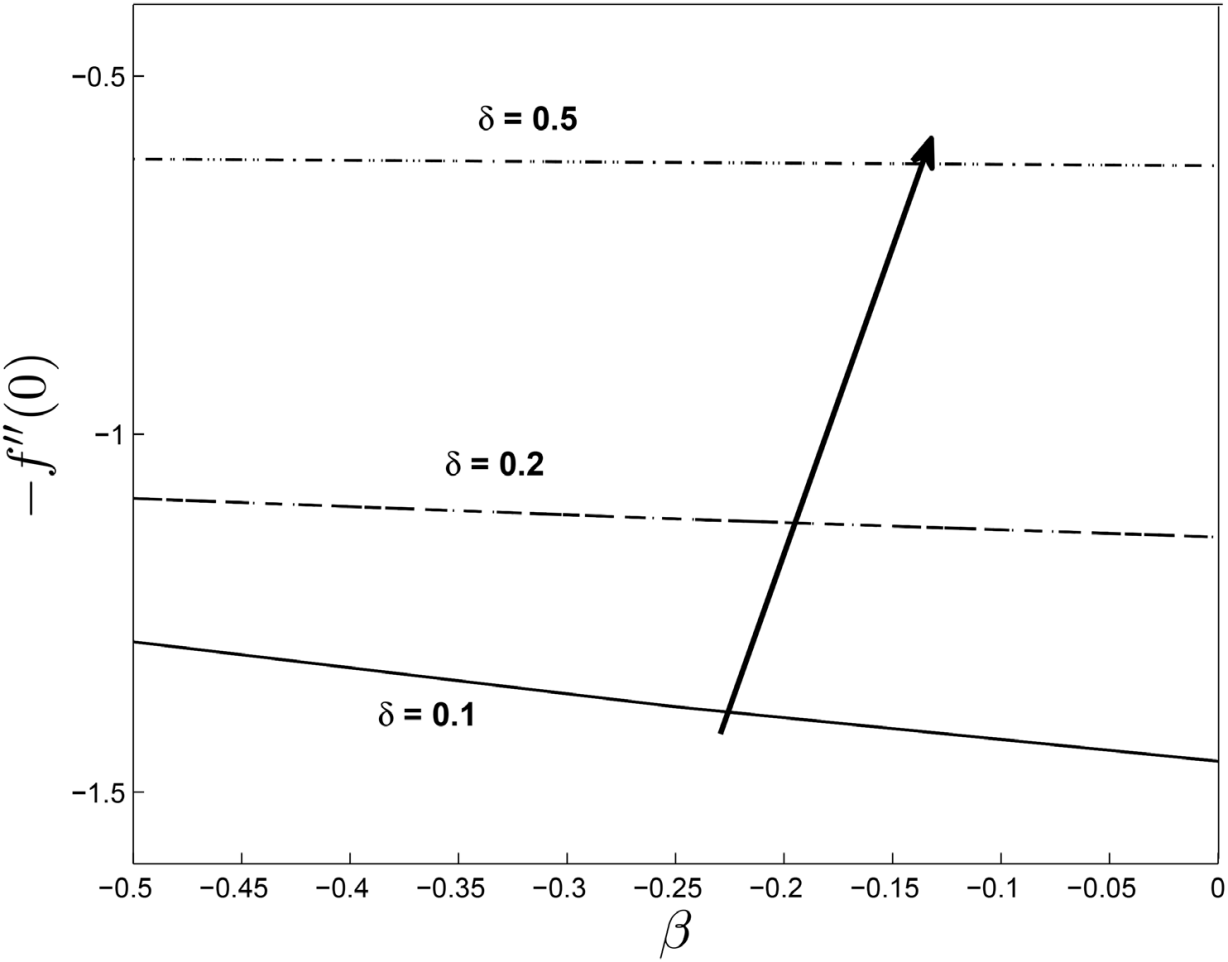
Effect of *δ* on Skin friction coefficients for *α* = −0.95, *Pr* = 0.72, *Nr* = 0.2, *Ec* = 1.0, *R* = 1.0, *L* = 1.0 and *ϵ* = 0.5.

Figs 19 and 20 show the variation in the skin friction coefficient −*f*′′(0) with respect to *β*. We observe that the skin friction coefficients decrease monotonically with increasing values of *β* and *M* in Fig 19 while the opposite is true in the Fig 20 for *β* and *δ*. The highest value of the skin friction is reached for smaller values of *β*.

**Fig 21:**
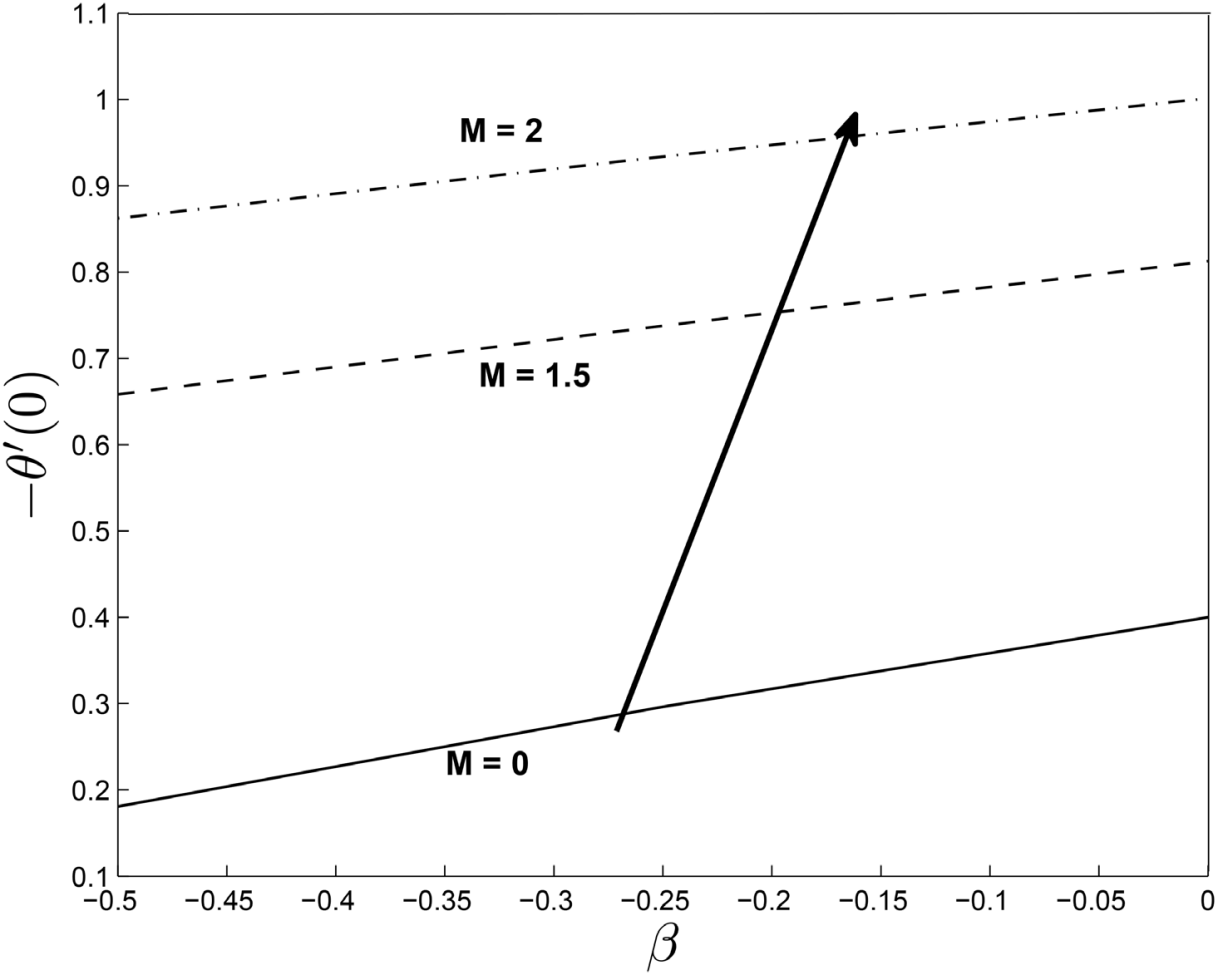
Effect of *M* on heat transfer coefficients for *α* = −0.95, *Pr* = 0.72, *Nr* = 0.2, *Ec* = 1.0, *R* = 1.0, *L* = 1.0 and *ϵ* = 0.5.

**Fig 22:**
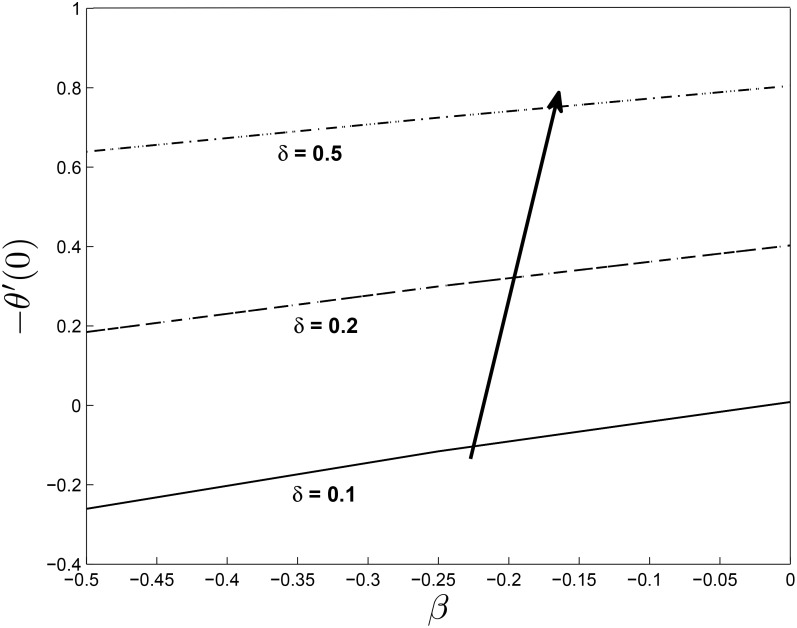
Effect of *δ* on heat transfer coefficients for *α* = −0.95, *Pr* = 0.72, *Nr* = 0.2, *Ec* = 1.0, *R* = 1.0, *L* = 1.0 and *ϵ* = 0.5.

Figs 21 and 22 display the dimensionless wall heat transfer rates −*θ*′(0) as a function of *β*. We observe that the wall heat transfer rate increases with increasing *β*, *M* and *δ*. The maximum value of the dimensionless wall heat transfer rates is achieved for large values of *β*.

## 6 Conclusion

An unsteady MHD axisymmetric stagnation-point flow over a shrinking sheet with temperature dependent thermal conductivity and thermal radiation and a Navier slip was investigated in this paper. The surface was assumed to shrink axisymmetrically in its own plane and the flow was permeated by a uniform magnetic field normal to the surface. The temperature profiles in the two cases of prescribed wall temperature and prescribed surface heat flux was shown to increase with the thermal radiation parameter, which in turn increases the thermal boundary layer thickness for both PST and PHF cases. This may be due to the fact that an increase in *N*_*r*_ induces a significant interaction between the fluid and the thermal boundary layer. It is clear that *f*′(*η*) increases with increasing magnetic parameter values *M* while *h*(*η*) decreases with the magnetic parameter. It can be concluded that for a shrinking sheet, the effect of non-alignment becomes less pronounced with enhanced magnetic parameter values. When slip occurs, the flow velocity near the sheet is no longer equal to the shrinking velocity at the sheet. Then with an increase in *δ* such slip velocity increases and consequently fluid velocity decreases for *h*(*η*) under the slip condition at the boundary.
